# *Hypericum empetrifolium* and *H. lydium* as Health Promoting Nutraceuticals: Assessing Their Role Combining In Vitro In Silico and Chemical Approaches

**DOI:** 10.1002/fsn3.70053

**Published:** 2025-03-31

**Authors:** Stefania Sut, Stefano Dall'Acqua, Giancarlo Angeles Flores, Gaia Cusumano, İsmail Koyuncu, Ozgur Yuksekdag, Carla Emiliani, Roberto Venanzoni, Paola Angelini, Selami Selvi, Evren Yildiztugay, Adriano Mollica, Eleonora Procino, Gregorio Peron, Lorenza Marinaccio, Gokhan Zengin

**Affiliations:** ^1^ Department of Pharmaceutical and Pharmacological Sciences University of Padova Padova Italy; ^2^ Department of Chemistry, Biology and Biotechnology University of Perugia Perugia Italy; ^3^ Botanic Garden “Giardino dei Semplici”, Department of Pharmacy “Gabriele d'Annunzio” University Chieti Italy; ^4^ Department of Medical Biochemistry, Faculty of Medicine Harran University Sanliurfa Turkey; ^5^ Department of Plant and Animal Production, Altınoluk Vocational School Balıkesir University Balıkesir Turkey; ^6^ Department of Biotechnology, Science Faculty Selcuk University Konya Turkey; ^7^ Department of Pharmacy University “G. d'Annunzio” of Chieti‐Pescara Chieti Italy; ^8^ SCM NutraceuticiUniversitari SRL Chieti Italy; ^9^ Department of Molecular and Translational Medicine University of Brescia Brescia Italy; ^10^ Department of Biology, Science Faculty Selcuk University Konya Turkey

**Keywords:** antioxidant, apoptosis, hyperforin, *Hypericum*, hyperoside, tyrosinase

## Abstract

*Hypericum* species are known for their ability to produce multiple classes of secondary metabolite and in this article the possible health‐promoting role of *H. empetrifolium* and *H. lydium* have been evaluated combining in vitro and in silico approaches. *H. empetrifolium* and *H. lydium* extracts were obtained using different solvents (ethyl acetate, aceton, aceton/water, and water) and the composition was compared using NMR and LC–MS based approaches. Myricetin‐3‐*O*‐glucoside, Kaemempferol ‐3‐*O*‐glucoside, and hyperopliphylirrin were present only in *H. empetrifolium.* Rutin, quercetin‐3‐*O*‐rhamnoside ant the triterpenoids oleanolic and ursolic acid were only detected in *H. lydium*. To establish a possible role against degenerative diseases antioxidant, antiradical activities of the extracts were studied and *H. empetrifolium* acetone/water and water extracts were the more active regarding these effects. The extracts were also evaluated for the inhibition of key‐enzymes involved in degenerative diseases namely cholinesterase and tyrosinase for CNS‐related pathologies and amylase for metabolic‐related diseases. Significant inhibition of acetylcholinesterase was observed for the extracts obtained with lipophilic solvents. The extracts were also studied for their possible antiproliferative activity on cell lines including tumor (DU‐145, A549, and MCF‐7) and non‐tumoral cells (HEK‐293) revealing moderate activities. *H. lydium* ethyl acetate showed late apoptotic (17.5%) and necrotic (46.6%) effects in the Annexin V/PI assay. Molecular docking was used to establish possible interaction of identified compounds with target enzymes and a good interaction between rutin and tyrosinase, myricetin‐7‐*O*‐glucoside and amylase, was reported. In conclusion, *Hypericum empetrifolium* and *H. lydium* thanks to their complex pattern of phytoconstituents and thanks to their significant antioxidant effects as well as with the ability to act on some key‐target enzymes involved in degenerative diseases can be considered a good vegetal source for the preparation of nutraceuticals and food supplements useful as health‐promoting products against oxidative stress‐related diseases such as diabetes, cancer, and Alzheimer's disease.

## Graphical abstract

The *Hypericum* can be considered a good vegetal source for the preparation of nutraceuticals and food supplements useful as health‐promoting products against oxidative stress‐related diseases such as diabetes, cancer, and Alzheimer's disease.

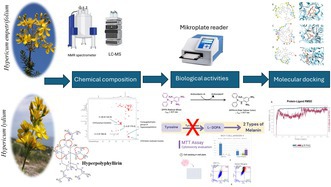

## 1 Introduction

Over the past decade, the demand for medicinal plants has been growing every day. Due to the concerns about using synthetics, medicinal plants, and their effects have become one of the most attractive topics on the scientific platform. The medicinal plants and their secondary metabolites are considered effective therapeutic agents in managing global health problems, including cancer, Alzheimer's disease, obesity, and diabetes mellitus (Açar et al. 2023; Şahin et al. 2023). Thus, more studies are needed to discover the potential of uninvestigated plant species instead of synthetic drugs.

The genus *Hypericum* L. belongs to the family Hypericaceae and includes around 500 species, making it one of the 100 largest genera within the angiosperm clade (Silva et al. 2021). The genus *Hypericum* exhibits a remarkable diversity in form and habitat, encompassing a wide range of species distributed globally. These species are found on every continent, thriving in environments ranging from temperate and subtropical regions to mountainous areas, with the notable exceptions of the Antarctic zone, polar regions, deserts, and low‐altitude tropical locales (Norman 2016; Nürk and Crockett 2011). Although most *Hypericum* species thrive in mild climates, they are also capable of adapting to humid or hot environments (Crockett and Robson 2011). The Mediterranean Basin is a key center of diversity for the genus, containing over 150 species within 22 sections, making it a hotspot for *Hypericum* biodiversity (Crockett and Robson 2011). Significant diversity is also found in Asia and the Americas, with many species being endemic. In Turkey, current literature identifies 109 taxa in 20 sections, of which 48 are endemic (Duman and Çakır‐Dindar 2020). Additionally, many species of *Hypericum* are cultivated for ornamental, medicinal, and timber purposes, further contributing to their global distribution and widespread use (Salinas et al. 2022). The etymology of the genus *Hypericum* comes from the Greek words “hyper” (above) and “eikon” (image), reflecting the ancient custom of hanging the plant's flowers above religious icons to ward off evil spirits. This tradition is also linked to the belief that *Hypericum* was used to protect against the “evil eye” and other negative influences. Over time, this practice became associated with Christian rituals, especially around the feast of St. John the Baptist, giving rise to the common name St. John's wort for some species of *Hypericum*. Several species of *Hypericum* are well known for their medicinal properties and have been used in traditional medicine across various cultures and have been part of therapeutic practices since ancient times (Kakouri et al. 2023; Tanaka and Kashiwada 2021). Notably, *Hypericum* species were mentioned in the *De Materia Medica* by Dioscorides (1st century AD), one of the most important pharmacological texts of antiquity (Beck 2005). Their use was also documented by Hippocrates and later by Nikolaos Myrepsos during the Medieval era (Valiakos et al. 2015, 2017). The medicinal value of *Hypericum* was recognized by prominent historical figures such as Theophrastus and Hippocrates, reflecting its long‐standing role in the history of medicine (Boga et al. 2021). Various species of *Hypericum* are traditionally used in folk medicine worldwide to treat a wide range of ailments, including mild depression, burns, diarrhea, wounds, pain, stings, bites, fevers, and infections (Caldeira et al. 2022). They are recognized for their properties as astringent, febrifuge, diuretic, anti‐inflammatory, analgesic, antispasmodic, and antidepressant agents. Additionally, *Hypericum* species are used to manage conditions such as migraines, epilepsy, neuralgia, sciatica, rheumatism, dyspepsia, parasites, and to stimulate bile flow (cholagogue) (Salinas et al. 2022). Furthermore, recent studies have highlighted the diverse biological activities of *Hypericum* species, including antitumor, antidepressant, pain‐relieving, anti‐inflammatory, antimicrobial, antioxidant, cytotoxic, and analgesic effects (Avato 2005; Marrelli et al. 2020, 2016; Silva et al. 2021; Zhang et al. 2020). Among these species, *Hypericum perforatum* stands out as the most researched, particularly for its role in treating mild to moderate depression (Whiskey et al. 2001; Zirak et al. 2019). In addition to its antidepressant properties, it has demonstrated antibacterial, antiviral, anti‐inflammatory, and spasmolytic effects, as well as potential applications in managing pain and conditions like trigeminal neuralgia (Assiri et al. 2017; Galeotti 2017). This makes *H. perforatum* one of the most popular and widely used herbal supplements. The medicinal value of *Hypericum* species is largely attributed to its bioactive compounds, particularly naphthodianthrones (such as hypericin, pseudohypericin, and isohypericin) and prenylated phloroglucinols (notably hyperforin and adhyperforin) (Kakouri et al. 2023). Additionally, the healing properties of *Hypericum* are linked to other phytochemicals, including flavonoids (rutin, hyperoside, isoquercitrin, quercitrin, quercetin, amentoflavone) and phenylpropanoids (chlorogenic acid) (Marrelli et al. 2016). Collectively, these molecules contribute to a wide array of biological activities, such as anti‐aging, anti‐inflammatory, antiviral, antibacterial, antidepressant, photodynamic, and antitumor effects. Although other *Hypericum* species have been utilized in traditional medicine, there have been relatively few studies conducted on them compared to *Hypericum perforatum* , which has been extensively researched for its medicinal properties (Boran and Ugur 2017). This work focuses on two species, *Hypericum lydium* Boiss and *Hypericum empetrifolium* Willd., found in Turkey. The coastal areas of western Turkey are considered the primary distribution zones of *H. empetrifolium*, which has been used for centuries in this region in traditional medicine for various therapeutic purposes (Robson 1967). In Turkey, decoctions of this plant, in addition to being used for dyeing fabrics, also had numerous healing applications, such as the treatment of kidney stones and gastric ulcers (Crockett et al. 2008). In addition to its well‐known healing and antimicrobial properties, *H. empetrifolium* has proven to be an effective anti‐inflammatory agent in numerous scientific studies (Boga et al. 2021). *H. lydium* is commonly known in Turkish as “sancı otu” (“cramp herb”) and “mayasıl otu” (“hemorrhoid herb”). In traditional Turkish folk medicine, this plant has been utilized for the treatment of a variety of ailments, including menstrual problems, stomach aches, wounds, hemorrhoids, and digestive issues (Boran and Ugur 2017). The present study considered the *H. empetrifolium* and *H. lydium* as potential vegetal sources for the development of nutraceutical and food supplements. To rationally proceed with the possible utilization of these plants a detailed chemical characterization is mandatory, and the exploration of possible composition of extracts obtained with different solvents is also necessary. In addition, due to the role of oxidative stress in different degenerative pathologies the capacity to scavenge radicals, reduce ions, chelate metals were studied. Furthermore, as a starting point the possible role on some enzymatic activities related to two groups of important degenerative diseases were considered. At first CNS diseases as Alzheimer, in which is well described the role of the acetylcholinesterase (AChE), butyrylcholinesterase (BChE). Another enzyme that has been considered is the tyrosinase, involved in Parkinson disease (Qi et al. 2024). As second group metabolic diseases mainly related to sugar level controls and in this case α‐amylase, and α‐glucosidase enzymes, have been evaluated as possible targets. TO have a preliminary study on the possible activities of the extract cytotoxic properties were evaluated on different cell lines.

## 2 Materials and Methods

### 2.1 Plant Collection

The botanical specimens were collected from Turkey (*H. empetrifolium*: Muğla, Karacasogut, 46 m; *H. lydium*: Akçapınar Location, Bozkır, Konya, 1230 m), Dr. Selami Selvi and Dr. Evren Yildiztugay conducted the taxonomic identification, and a voucher specimen was preserved in the herbarium of Balikesir (Voucher number: SV 3760 for *H. empetrifolium*) and Selcuk University (Voucher number: EY‐3103 for *H. lydium*). The aerial parts were segregated, dried in the shade at ambient temperature, pulverized, and thereafter stored away from light.

### 2.2 Plant Extract Preparation

The extraction procedure included four solvents: ethyl acetate, acetone, a 70% acetone/water mixture, and water. Each 10 g sample was macerated with 200 mL of ethyl acetate, acetone, and a mixture of acetone and water for 24 h at ambient temperature. The aqueous extract was prepared by infusing 10 g of plant material in boiling water for 15 min. Organic solvents were removed via evaporation under low pressure, and the aqueous extract was subjected to freeze‐drying.

### 2.3 Assay for Total Phenolic and Flavonoid Contents

Total phenolics and flavonoids were quantified according to the procedures outlined by (Slinkard and Singleton 1977). Gallic acid (GA) and rutin (RE) used as reference standards in the studies, with results expressed as gallic acid equivalents (GAE) and rutin equivalents (RE).

### 2.4 Chemical Composition

NMR analyses were obtained on a Bruker Avance III spectrometer operating at 400 MHz, standard bruker pulse sequences were used for the acquisition of ^1^H, HSQC‐DEPT, COSY, TOCSY experiments. Samples were dissolved in appropriate deuterated solvent (deuterated chloroform or deuterated methanol‐Sigma Aldrich) and used for the analysis. Agilent 1290 UPLC system equipped with 1290 series Diode Array was used as a chromatograph coupled with the Quadruple time of Flight (QTOF). After the column, the flow was split with a passive T junction and the liquid was sent to a diode array or mass spectrometer. The Agilent 6530 QTOF was used as a detector. The instrument is equipped with a Jet Stream source and was operating in positive ion mode. During acquisition, MS was calibrated using the calibration mixture of Agilent. A Agilent SB C18 (3 × 100 mm; 1.7 μm) column was used as stationary phase. The eluents were (A) water 0.1% formic acid and (B) acetonitrile 0.1% formic acid. The flow rate was set to 0.5 mL/min and gradient started 90% of A then reach 85% B in 14 min and then reached 90% B in 20 min and remained isocratic up to 24 min. For the Low‐Resolution MS was acquired using an Agilent 1290 chromatograph, equipped with diode array and coupled with the Varian ion trap MS500 operating in ESI mode acquiring data in negative ion mode. As column an Agilent XDB C18 (3 x 150) was used, and eluents were (A) water 0.1% formic acid and (B) acetonitrile 0.1% formic acid. Flow rate was 0.4 mL/min and gradient started with 90%A and reached 5% A in 15 min. The instrument was operating in turbo data depending scan allowing the generation of fragmentation tree for the most abundant compounds. For compound.

For the compound identification, matching of the HR‐MS and calculation of molecular formula, matching of the MS and fragmentation data obtained in the Ion Trap as well as UV spectrum compatibility, and when available the comparison with the reference standard. Due to the non‐availability of some reference compounds, a tentative identification was obtained matching MS data, UV spectrum, and previous identification from references was adopted. MS parameters were needle voltage 4500 V, nebulizer gas pressure 25 psi, drying gas pressure 15 psi, drying gas temperature 260°C, spray chamber temperature 50°C, capillary voltage 80 V and RF loading 80%. For quantification, chlorogenic acid, quercetin‐3‐*O*‐glucoside, quercetin‐3‐*O*‐galactoside (hyperoside), hyperforin and hypericin were used. Standard solutions were prepared in methanol: water (50:50) for chlorogenic acid, methanol for quercetin‐3‐*O*‐glucoside, quercetin‐3‐*O*‐galactoside and hyperforin and methanol: DMSO (50:50) for hypericin, respectively. Hyperpolyphillirin was quantified using hyperforin as reference compound. Standard solutions were prepared at four different concentrations in a range of 50–1 μg/mL, and calibration curves were calculated.

### 2.5 Assays for In Vitro Antioxidant Capacity

In accordance with the methodologies detailed in our previous publication (Grochowski et al. 2017), various antioxidant tests were carried out. The outcomes were represented as milligrams of Trolox equivalents (TE) per gram for the DPPH, ABTS radical scavenging, CUPRAC, and FRAP tests. In millimoles of Trolox equivalents (TE) per gram of extract, the phosphomolybdenum (PBD) test examined antioxidant potential, and in milligrams of disodium edetate equivalents (EDTAE) per gram of extract, the metal chelating activity (MCA) was determined.

### 2.6 Inhibitory Effects Against Some Key Enzymes

In accordance with the established protocols (Grochowski et al. 2017), experiments on enzyme inhibition were performed on the samples. Acarbose equivalents (ACAE) per gram of extract was used to measure the activities that inhibit amylase and glucosidase, whereas milligrams of galanthamine equivalents (GALAE) per gram of extract was used to examine the inhibition of acetylcholinesterase (AChE) and butyrylcholinesterase (BChE). The amount of tyrosinase inhibition for each gram of extract was measured in milligrams of kojic acid equivalents (KAE).

#### 2.6.1 Cytotoxic Properties

##### 2.6.1.1 Cell Culture

DU‐145 (Prostate Carcinoma), MCF‐7 (Breast Adenocarcinoma), A549 (Lung Adenocarcinoma) and HEK‐293 (Human Embryonic Kidney) cell lines were obtained from ATCC, frozen into liquid nitrogen and stored until ready for use, then cultured in DMEM‐F12 or RPMI‐1640 media containing 10% FBS and antibiotics (100 μg/mL streptomycin and 100 IU/mL penicillin) at 37°C with 5% CO_2_ in a humidified incubator.

##### 2.6.1.2 Cell Viability Assay

MTT assay (3‐(4,5‐Dimethylthiazol‐2‐yl)‐2,5‐Diphenyltetrazolium Bromide) was done to determine the cytotoxicity of the extracts. Cells (DU‐145, MCF‐7, A549 and HEK‐293) at a density of 1 × 10^4^ cells per well in sterile plates with 96 wells were placed overnight. After 24 h, the media were replaced by extracts at concentrations of 0, 2.5, 5, 10, 25, 50, 100 and 200 μg/mL for an additional incubation period of 24 h Each well received 10 mL of MTT solution (0.5 mg/mL) and after keeping it for the next 4 h, the media were replaced with 100 μL of DMSO. The OD570–OD690 nm absorbance was measured using a Thermo Multiskan GO plate reader, and IC_50_ values were calculated from the data.

##### 2.6.1.3 Apoptotic Effect on MCF‐7 Cancer Cell With Acridine Orange/Ethidium Bromide (AO/EB) Staining

Apoptosis was detected morphologically in treated MCF‐7 cells (100 μg/mL ethyl acetate extract of *H. lydium*). The treated cells were washed with PBS, fixed in 70% ethanol and then washed in distilled water after incubation. Subsequently, the cells were stained with Acridine Orange/Ethidium Bromide working solution (Cat No./ID: A6014‐E1510, Sigma Aldrich, Germany) and images were taken using fluorescence microscopy.

##### 2.6.1.4 Apoptotic Effect on MCF‐7 Cancer Cell With Annexin V

FITC Annexin V Apoptosis Detection Kit I commercial (BD Biosciences, New Jersey, USA) was used according to the manufacturer's instructions. Cell MCF‐7 was placed into 6‐well plates (5 × 10^5^ cells/well) and then incubated for 24 h, and then post‐cell plating, the ethyl acetate extract of *H. lydium* with a concentration of 100 μg/mL was added followed by another 24 h incubation. Trypsinized cells were diluted in 1× binding buffer and resuspended at 1 × 10^6^ cells/tube. The tubes were incubated at room temperature for 15 min. Subsequently, 5 μL of fluorochrome‐conjugated Annexin V and 5 μL of Propidium Iodide dyes were added. After the cells, 100 μL of 1× binding buffer were added; the tubes were centrifuged at 1200 rpm and incubated for 5 min. Finally, the cells were analyzed by flow cytometry (BD. Via, New Jersey, USA).

#### 2.6.2 Molecular Modeling

##### 2.6.2.1 Proteins and Ligands Preparation

The crystal structures of the enzymes were downloaded from the protein RCSB database (https://www.rcsb.org/): Amylase (PDB ID: 1VAH), AChE (PDB ID: 4X3C), BChE (PDB ID: 4BDS), Glucosidase (PDB ID: 3AXI), Tyrosinase (PDB ID: 2Y9X). The raw crystallographic proteins were prepared by the PrepWizard module of Maestro Schrödinger (2017). The missing side chains were added by Prime and the protonation state was estimated by PropKa at pH 7.0. The proteins were next minimized using OPLS4 force field following a well‐established protocol published previously (Bender et al. 2023; Mamadalieva et al. 2021). Computational experiments were performed for selected compounds: 3‐p‐CoQA (**785**), 5‐CQA (**633**), 4‐CQA (**666**), Myricetin‐7‐*O*‐glucoside (**443**), Rutin (**805**), Quercetin‐3‐*O*‐galactoside (**643**), Quercetin‐3‐*O*‐rhamnoside (**915**), Quercetin (**343**), Hyperpolyphyllirin (RiH), BisApigenin (**856**), Hyperforin (**298**). These compounds were downloaded from PubChem database, except for RiH, which was drawn using Maestro. They were all prepared by LigPrep tool embedded in Maestro, using Epik at pH 7.4 and OPLS4 force field. The docking grid was generated using the Glide module of Maestro, it was centered on the crystallographic ligand and then the glide was used for docking experiments (Bender et al. 2023; Mamadalieva et al. 2021).

### 2.7 Molecular Docking and Molecular Dynamics

The prepared ligands were docked to each protein prepared by Maestro. Briefly, the docking experiments were carried out by using Glide, firstly using Standard Precision (SP) scoring function. The ligand was set to be flexible, whereas the protein was kept rigid. The best pose generated from the first step was submitted to Molecular Docking using eXtra Precision scoring function (XP), following the previously reported protocol used by our research group (Sinan et al. 2023; Stefanucci et al. 2021).

Since the tested compounds appear to have the highest biological activity on Tyrosinase, Molecular Dynamics experiment was carried out on the best pose obtained in Molecular Docking with XP function of Tyrosinase‐805Rutinsystem. The system builder instrument in Desmond was used for the preparation of the receptor–ligand complex. The entire system was centered by an orthorhombic box, which was saturated with water molecules by setting the TIP4P aqueous solvent model, to recreate physiological conditions. The NaCl salt was added in the “Ions” section to neutralize and the OPLS3 force field was set. The resulting system, displayed in the Workspace, was loaded in the “Molecular Dynamics” panel, belonging to Desmond. For Tyrosinase–805Rutin system, the overall simulation time was 20 ns, with an approximate number of frames equal to 200. For the ensemble option, which represents the macroscopic conditions of the system, the statistical set NPT (ensemble isothermal‐isobaric), characterized by constant values of the number of particles, pressure, and temperature. For the MD experiment, the following parameters have been set: (a) temperature at 309 K and (b) pressure at 1.01325 bar.

### 2.8 Statistical Analysis

Experiments were performed in triplicate, and differences between the extracts were compared using One‐way ANOVA (by Tukey's assay) and GraphPad Prism (version 9.2) was used for the analysis. The *p* value of < 0.05 was deemed to be statistically significant. Correlation analysis was performed by considering chemical and bioactivity data. Calculated amounts of identified metabolites and bioactivity results obtained from antioxidant and enzyme inhibition assays were correlated by Pearson's analysis performed with Metaboanalyst v. 6 (https://www.metaboanalyst.ca/). Before analysis, data were processed by log‐transformation and Pareto scaling. The results were graphically displayed by a heatmap, where positive correlations were indicated by red color and negative ones by blue color. Correlation coefficients (Pearson's *r*) were comprised between +1 and −0.9.

## 3 Results and Discussion

### 3.1 Total Phenolic and Flavonoid Content

The total phenolic and flavonoid content in the tested extracts was evaluated using the Folin–Ciocalteu assay. As shown in Table 1, for *H. empetrifolium*, the highest phenolic compound content was found in the water and acetone/water extracts, with values of 99.84 ± 1.23 mg GAE/g and 98.23 ± 2.09 mg GAE/g, respectively. These were followed by the acetone extract (47.23 ± 1.25 mg GAE/g) and the ethyl acetate extract (38.05 ± 2.33 mg GAE/g). Regarding flavonoid content, a similar extraction pattern was observed, with values of 44.64 ± 0.22 mg RE/g for the water extract, 41.32 ± 0.32 mg RE/g for acetone/water, 24.12 ± 0.46 mg RE/g for acetone, and 21.09 ± 0.63 mg RE/g for ethyl acetate. For *H. lydium*, the highest phenolic content was found in the acetone/water extract, with a value of 93.11 ± 1.15 mg GAE/g, followed by the water extract (68.47 ± 0.36 mg GAE/g), acetone (53.37 ± 0.84 mg GAE/g), and ethyl acetate (28.62 ± 0.46 mg GAE/g). In terms of flavonoid content, the acetone/water extract showed the highest value (52.49 ± 0.46 mg RE/g), followed by acetone (50.22 ± 0.31 mg RE/g), water (36.34 ± 0.13 mg RE/g), and ethyl acetate (29.08 ± 0.34 mg RE/g). The results indicate that all extracts of *H. empetrifolium* were richer in phenolic compounds compared to *H. lydium*, with ranges from 38.05 ± 2.33 to 99.84 ± 1.23 mg GAE/g and 28.62 ± 0.46 to 93.11 ± 1.15 mg GAE/g, respectively. However, the comparison of flavonoid content revealed a different trend. The TFC values for *H. empetrifolium* ranged from 21.09 ± 0.63 to 44.64 ± 0.22 mg RE/g, whereas for *H. lydium* they ranged from 29.08 ± 0.34 to 52.49 ± 0.46 mg RE/g. Our results are consistent with those of Eruygur et al. (2019), who found values of 68.93 ± 5.29 mg GAE/g for total phenolic content (TPC) and 4.97 ± 4.56 mg RE/g for total flavonoid content (TFC) for aqueous extracts of *H. lydium*. The total phenolic content had previously been investigated by Boran and Ugur (2017), who evaluated the total phenolic content of an ethanolic extract of *H. lydium*, with a value of 135 ± 1.11 mg GAE/g extract. Even earlier, the phenolic content of *H. lydium* had been assessed by Can et al. (2012), who reported a gallic acid equivalent (GAE) content of methanol extracts of *H. lydium* fruits and flowers as 1.61 ± 0.17 and 1.92 ± 0.22 mg per gram of dry weight, respectively. However, the results of these two studies (Boran and Ugur 2017; Can et al. 2012) are not directly comparable with those of our study, as the extracts were prepared in different ways and with different solvents. In the study by Boga et al. (2021), the total phenolic and total flavonoid contents of the ethanolic extracts from the aerial part (HEA) and root (HER) of *H. empetrifolium* were determined in micrograms of pyrocatechol equivalents (PEs) and micrograms of quercetin equivalents (QEs), respectively. The reported values were 50.12 ± 0.97 (μg PEs/mg extract) and 7.23 ± 0.20 (μg QEs/mg extract) for HEA; 26.28 ± 0.73 (μg PEs/mg extract) and 2.33 ± 0.07 (μg QEs/mg extract) for HER. Again, these results are not directly comparable to those found in our study as the experiments were conducted using different methods. Our study, however, highlights that the extracts of these two species, especially acetone/water and water extracts, are rich in phenols and flavonoids, which is reflected in their strong antioxidant capacity, as shown in Table 3.

**TABLE 1:** Total phenolic and flavonoid contents in the tested extracts.

Species	Solvents	TPC (mg GAE/g)	TFC (mg RE/g)
*Hypericum empetrifolium*	EA	38.05 ± 2.33^f^	21.09 ± 0.63^h^
	Acetone	47.23 ± 1.25^e^	24.12 ± 0.46^g^
	Acetone/Water	98.23 ± 2.09^a^	41.32 ± 0.32^d^
	Water	99.84 ± 1.23^a^	44.64 ± 0.22^c^
*Hypericum lydium*	EA	28.62 ± 0.46^g^	29.08 ± 0.34^f^
	Acetone	53.37 ± 0.84^d^	50.22 ± 0.31^b^
	Acetone/Water	93.11 ± 1.15^b^	52.49 ± 0.46^a^
	Water	68.47 ± 0.36^c^	36.34 ± 0.13^e^

*Note:* Values are reported as mean ± SD of three parallel measurements. Different letters indicate significant differences between tested extracts (*p* < 0.05).

Abbreviations: GAE, gallic acid equivalents; RE, rutin equivalents.

### 3.2 Determination of the Phytochemical Profile

For the determination of the composition of the extracts NMR approach was used for the *H. empetrifolium* extracts to establish which are the different detectable classes of phytoconstituents. *H. empetrifolium* extracts were obtained with solvents with different polarity, starting from ethyl acetate as most apolar, then using acetone, a mixture acetone water and as more polar water. The different physicochemical properties of the solvents are related to their ability to penetrate the vegetal material as well as to their ability to bring in solution and extract the plant phytochemicals. The NMR analysis shows differences ascribable to the different polarity of extracted constituents.

Ethyl acetate fractions show a large presence of lipid derivatives and the main resonances ascribable to these constituents are summarized in the Supporting Information in the Figures S1 and S2 and Table S1. Signals of fatty acids support the presence of unsaturated and saturated constituents. The spin system ascribable to fatty acid show coupling from the signal at δ 0.89 ascribed to a terminal methyl group with 1.27–1.31 from these latter with 1.67 (ascribable to CH2 in alpha to carbonyl groups) and 2.05 assigned to carbon chain of unsaturated fatty acids (CH2 in alpha to double bonds). Furthermore, signals ascribable to glycerol and double bonds are detected and carbon resonances are collected in HSQC while long range correlations in HMBC (see Figures S2–S4). Also signals suggesting the presence of other aliphatic compounds are present but are in large part superimposed to fatty acids. Comparing the H‐NMR of the acetone extract we can observe a clearer aliphatic region and the analysis of 2D spectra allows the identification of phloroglucinol diagnostic signals in particular the methyl groups and the sp2 (see Figures S4–S9).

**FIGURE 1:**
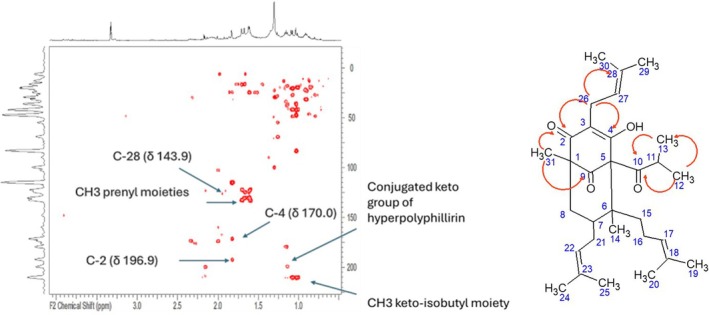
Hyperpolyphyllirin structure, red arrow indicates the diagnostic HMBC correlations that are indicative for the structure elucidation.

Starting from the sp2 protons of prenyl moieties (see Table S1) and the methyl groups of phloroglucinol derivatives we can detect some part of the main structure and suggest the presence of a main derivative. The sp2 CH are detected as two group of signals at δ 5.02 and 5.04 showing in HSQC‐DEPT correlation with carbon resonances at δ 122.1 and 124.3, respectively. These CH show in COSY spectrum coupling with singlets at δ 1.60, 1.62, 1.67, and 1.70 ascribed to methyl group of prenyl units (see Table S1). Also signals supporting the presence of CH_3_ keto‐isobutyl moiety are observed namely the doublets at δ 1.05 and 1.08 coupled with the CH at δ 2.16–40.5. The two methyl groups of the keto‐isobutyl unit show HMBC correlations with keto carbon at δ 210.0. Furthermore, another quaternary methyl group (δ 1.60–17.9) superimposed to the previous revealed an HMBC with conjugated keto function (δ 198.0) and can be ascribed to the methyl 31 of the structure of hyperpolyphillirin. Also, other diagnostic correlations can be observed from the CH_2_–20 (δ 1.31–22.7) that show diagnostic correlations with the conjugated keto group C‐2 (δ 196.9), the oxygenated C‐4 (δ 170.0) and the quaternary sp2 C‐28 (δ 143.9). All this information can support the presence of hyperpolyphillirin or similar derivative bearing a methyl group nearby a conjugated keto group and a non‐conjugated one. A figure summarizing the key HMBC correlations is reported in Figure 1.

Another class of compounds that appear to be significantly present in the acetone and in the more hydrophilic fractions are the hydroxycinnamic acids. Signals ascribable to the trans olefinic moiety are clearly detected in acetone/water and water extracts (see Table S2).

Although preliminary this information has been used as a guide in the further step of phytochemical characterization and comparison of the two plants that has been obtained combining LC‐DAD‐MS^n^ and LC‐ESI‐QTOF.

As a first results data from the diode array were evaluated and numerous peaks showing significant UV absorption presenting maximum in the range 340–360 nm and typical shape of flavonoids were identified. Furthermore, a series of peaks presenting UV max in the range 320–330 nm were observed suggesting the presence of hydroxycinnamic derivatives. On the other hand, no significant peaks were observed showing maximum absorption in the range 530–560 nm indicating at least non detectability by the diode array of hypericin derivatives. Some peaks ascribable to flavonols are identified and further information were obtained from the mass spectrometry combining the data from multiple stage low‐resolution Ion Trap (MS^n^) and high‐resolution quadrupole‐time of flight working with increasing collision energy (MS^e^).

Significant information can be obtained considering the presence of different isomeric caffeoyl‐quinic acid identified on the basis of their UV maximum at around 330 nm and thanks to the HR‐MS showing the presence of different derivatives (see Table 2). The MS^n^ offer the opportunity to discriminate the 3, 4, and 5‐caffeoyl‐quinic (CQA) as previously described by Clifford et al. (2003) and we could observe different isomers in the obtained extracts. Also, some dicaffeoyl quinic acid were identified (Clifford et al. 2003) and these compounds are effectively extracted especially with acetone water and water solvents. Such phenolics are the most abundant constituents in the more hydrophilic extracts. In minor extent several flavonoid derivatives were identified by comparison with the literature (Fabre et al. 2001; Kachlicki et al. 2016) and reference compounds. Quercetin, myricetin, luteolin, and kaempferol derivatives were identified, also quercetin, luteolin were identified as aglycone and with bis‐apigenin were especially found in acetone extract. Main flavonoid is hyperoside that is efficiently extracted in aqueous media namely aceton/water and water compared to ethyl acetate.

**TABLE 2:** Chemical composition of the tested *Hypericum* extracts (mg/g).

		M‐H	Fragments	*H. hempetifolium*				*H. lydum*			
				Ethyl acetate	Aceton	Aceton/Water	Water	Ethyl acetate	Aceton	Aceton/Water	Water
Quinic acid	C7H11O6	191,0546		0.01	0.02	1.25	1.62	0.40	0.32	5.30	4.65
Protocatecuic acid hexoside	C13H15O9	315,07724	153	0.41	0.46	3.38	1.86	—	—	—	—
**Flavan‐3‐ols**											
PAC B2	C30H25O12	577,269	461,425,409,289	0.05	0.67	1.88	0.41	0.05	0.08	0.31	0.74
Catechin	C15H14O6	289,0712		0.08	0.33	0.55	0.1	0.33	0.61	0.13	0.05
Dactylifric acid (Shikimoyl quinic acid)	C16H16O8	335,07198	293,253,191,161,135	0.65	0.85	0.32	2.74	0.46	0.24	0.28	1.28
PAC‐B2 hexoside	C36H35O17	739,16,395	677,288,451,289	0.87	1.42	2.14	0.90	0.16	0.21	0.60	0.26
**Hydroxycinnamic derivatives**											
3‐CQA	C16H17O9	353,08932	191 (BP) 179,173,127 85	0.35	0.90	2.40	1.73	0.01	0.67	6.51	8.06
3‐p‐CoQA	C16H17O8	337,0847	191 (BP) 161,119	1.90	3.50	72.27	61.40	0.06	0.22	2.64	4.31
5‐CQA	C16H17O9	353,0873	191 (BP) 173	2.44	9.50	74.52	68.85	0.00	0.27	1.63	2.57
4‐CQA	C16H17O9	353,08804	191,173,127 85	0.60	0.45	13.60	14.55	0.27	0.21	3.80	3.78
Caffeic acid	C9H7O4	179,03359	135,1	0.69	0.11	3.02	0.73	—	—	—	—
Methoxy‐2,3,4‐trihydroxycinnamic acid	C10H9O5	209,08757	194,166,139,111	0.81	0.08	2.80	0.13	—	—	—	—
4‐p‐CoQA	C16H17O8	337,0847	1,91	0.13	0.19	3.99	1.05	0.11	0.10	1.60	1.71
5‐Feruloyl‐QA	C17H19O9	367,10,389	191,173,127 85	0.08	0.20	4.42	0.43	0.64	0.07	1.14	26.16
3,5‐DCQ	C25H24O12	515,12,344	353,191,179,173,135	0.20	0.35	2.23	2.15	—	—	—	—
4,5‐DCQ	C25H24O12	515,12,344	353,173,179,191	0.06	0.12	3.93	2.91	—	—	—	—
Rosmarinic acid	C18H17O8	359,085	161,133,115	0.63	2.87	2.71	2.35	—	—	—	—
**Flavonoids**											
Myricetin‐3‐*O*‐glucoside	C21H19O13	493,0663	317,298,259,192,179	0.18	0.38	1.58	2.24	—	—	—	—
Kaempferol‐3‐*O*‐glucoside	C21H19O11	447,0815	285	0.28	0.24	1.23	3.22	—	—	—	—
Myricetin‐7‐*O*‐glucoside	C21H19O13	479,0831	317,316,270,179	0.70	7.51	8.78	1.10	0.62	0.69	0.63	1.80
Quercetin‐glucuronide‐hexoside	C27H27O18	639,12,035	549,477,301	0.27	4.00	4.82	1.66	—	—	—	—
Rutin	C27H30O16	609,1455	301,271,255,179	—	—	—	—	9.06	15.50	25.74	13.82
Quercetin‐3‐*O*‐glattoside (iperoside)	C21H19O12	463,1005	301,271,255,179	0.45	0.81	52.48	59.87	1.46	3.24	38.50	46.69
quercetin‐3‐*O*‐glucuronide	C21H17O13	477,0773	301,271,179,151	0.09	1.38	5.12	4.53	—	—	—	—
Acetyl‐hyperoside	C23H22O13	505,1019	463 301,271,255,179 151	0.99	0.99	4.75	4.92	—	—	—	—
Quercetin‐7‐*O*‐pentoside	C20H18O11	433,07998	300,255,179	1.33	0.76	2.15	2.22	1.53	0.16	0.22	25.67
Quercetin 3 O rhamnoside	C21H20O11	447,09267	301,283,271,255,179,151	No	—	—	—	1.17	29.60	43.72	0.69
Kaemferol‐7‐*O*‐rhamnoside	C21H20O10	431,0977	285,267,257,255,241,229	No	—	—	—	0.12	1.23	1.15	0.78
Luteolin	C15H9O6	285,0468	240,217,201,175,151	2.68	1.45	24.60	1.50	0.11	0.09	0.13	2.89
Quercetin	C15H9O7	301,04311	273,225,149 252	1.88	7.94	15.50	1.34	0.25	0.60	0.24	2.73
BisApigenin	C30H17O10	537,08387	493,443,385	7.23	14.23	16.81	0.74	6.60	1.83	0.08	1.80
		**M + H**									
Dihydrolaempferol	C15H12O6	289,07073	274,228	0.15	0.62	0.85	0.34	0.12	0.67	0.96	0.25
Dihydroisorhamnetin	C16H14O7	319,08105		0.13	0.42	0.64	0.25	0.13	0.72	0.99	0.31
**Phloroglycinols**											
Hyperpolyphilirrin	C31H45O4	481,28,119	411,2	18.02	5.38	13.20	0.85	—	—	—	—
Hyperpolyphilirrin isomer	C31H45O4	481,2809	411,2	13.20	4.56	16.50	0.71	—	—	—	—
Hyperfirin	C30H43O4	467,32,081	379,337,296,207	14.50	2.99	18.18	0.12	1.49	0.18	0.06	0.60
**Triterpene**											
Oleanolic acid	C30H47O3	455,35,419		—	—	—	—	4.53	2.23	2.65	0.20
Ursolic acid	C30H47O3	455,35,419		—	—	—	—	4.09	2.34	2.53	0.34
**Names**			**Fragments**								
6‐Hydroxyluteolin	C15H10O7	303,05059	257,229,153	0.15	0.19	0.10	0.05	0.22	0.29	0.11	0.08
Gallocatechin‐p‐coumarate	C24H20O9	453,11,819		0.42	0.44	0.16	0.18	0.33	0.35	0.16	0.15

Extracts from *H. empetrifolium* and *H. lydium* were analyzed using UPLC coupled with high‐resolution MS (QToF). The analysis revealed a diverse array of metabolites in the extracts, identifying several compounds through MS and MS^e^ data, complemented by comparisons with Ion trap (MS^n^) fragmentation and published literature. The extraction yields varied significantly across solvents and species. For *H. empetrifolium*, acetone–water extract yielded the highest quantity of identified metabolites (379.30 mg/g), followed by water (247.20 mg/g), acetone (74.29 mg/g), and ethyl acetate (71.69 mg/g). In contrast, *H. lydium* showed a different pattern, with water extract yielding the highest amount (146.36 mg/g), followed by water (128.97 mg/g), acetone (55.18 mg/g), and ethyl acetate (24.17 mg/g). The results of the extraction efficiency highlighted the species‐specific nature of metabolite solubility and the importance of solvent selection in phytochemical studies.

Considering the differences between the two species we can observe that the chlorogenic acid derivatives, such as 3‐*p*‐CoQA, 5‐CQA, and 4‐CQA, were found in much higher concentrations in *H. empetrifolium* compared to *H. lydium*. In *H. empetrifolium*, 3‐*p*‐CoQA and 5‐CQA were particularly abundant in the acetone–water extract (72.27 and 74.52 mg/g, respectively) and the water extract (61.40 and 68.85 mg/g, respectively) also 4‐CQA was present in significant amount in the water (14.55 mg/g) and acetone–water (13.60 mg/g) extracts. This is also related to the high hydrophilicity of these compounds. In contrast, *H. lydium* showed much lower concentrations of the same compounds, with the highest levels of 3‐*p*‐CoQA and 5‐CQA found in the water extract (4.31 and 2.57 mg/g, respectively), and the highest level of 4‐CQA in the acetone–water extract (3.80 mg/g).

Myricetin‐7‐*O*‐glucoside was identified in both species, with *H. empetrifolium* showing higher concentrations, particularly in the acetone–water (8.78 mg/g) and acetone (7.51 mg/g) extracts. *H. lydium* had lower but consistent levels across all extracts, with the highest concentration in the water extract (1.80 mg/g). Both species contained significant amounts of quercetin‐3‐*O*‐galactoside (hyperoside), but *H. empetrifolium* showed higher concentrations, especially in the water (59.87 mg/g) and acetone–water (52.48 mg/g) extracts. *H. lydium* also had considerable amounts, with the highest levels in the water (46.69 mg/g) and acetone–water (38.50 mg/g) extracts. *H. empetrifolium* showed higher levels of quercetin, with the highest concentration in the acetone–water extract (15.50 mg/g), followed by the acetone extract (7.94 mg/g). *H. lydium* had lower quercetin levels, with the highest amount in the water extract (2.73 mg/g).

Both species contained bisapigenin, but *H. empetrifolium* showed higher concentrations, particularly in the acetone–water (16.81 mg/g) and acetone (14.23 mg/g) extracts. *H. lydium* had lower levels, with the highest concentration in the ethyl acetate extract (6.60 mg/g). Hyperforin, also reported by Kakouri et al. (2023) in *Hypericum* spp., was present in both species in our study. *H. empetrifolium* contained higher levels of hyperforin compared to *H. lydium*, with the highest concentration found in the acetone–water extract of *H. empetrifolium* (18.18 mg/g), followed by the ethyl acetate extract (14.50 mg/g). *H. lydium* showed much lower levels, with the highest concentration in the ethyl acetate extract (1.49 mg/g).

Trying to differentiate the two species from phytochemical point of view we noticed that rutin was only detected in *H. lydium*, with the highest concentration in the acetone–water extract (25.74 mg/g), followed by the acetone extract (15.50 mg/g). Also, quercetin 3‐*O*‐rhamnoside was only detected in *H. lydium* extracts, with notably high concentrations in the acetone–water (43.72 mg/g) and acetone (29.60 mg/g) extracts. The oleanolic and ursolic acid were only present in *H. lydium* with higher amount in ethyl acetate extract.

The hyperpolyphyllirin was exclusively found in *H. empetrifolium* extracts, with the highest concentration in the ethyl acetate (18.02 mg/g), followed by the acetone–water (13.20 mg/g) also rosmarinic acid, 3,5‐DQA, and 4,5‐DQA were only found in *H. empetrifolium* extracts. In summary, *H. empetrifolium* extracts generally showed higher concentrations of the analyzed compounds, especially chlorogenic acid derivatives, quercetin, hyperpolyphyllirin, and hyperforin. *H. lydium*, on the other hand, was characterized by the presence of rutin and quercetin‐3‐*O*‐rhamnoside, which were not detected in *H. empetrifolium*. Furthermore, it can be hypothesized that the prevalence of some phenolic compounds, found in greater quantities in *H. empetrifolium*, particularly in the acetone/water and water extracts, could contribute, at least in part, to the strong antioxidant activity demonstrated by these extracts.

### 3.3 Antioxidant Effects

Scavenging, reducing, and metal chelating assays are essential tools for evaluating the antioxidant capacity of plant extracts. In this study, the antioxidant potential of different extracts from *Hypericum empetrifolium* and *Hypericum lydium* was assessed using DPPH and ABTS assays for scavenging activity, CUPRAC, FRAP, and phosphomolybdenum assays for reducing capacity, and a metal chelating assay. As shown in Table 3, the acetone/water and water extracts exhibited the highest quenching abilities for both *Hypericum* species in the scavenging assays. For *H. empetrifolium*, the best extract in the DPPH assay was acetone/water, with a value of 392.71 ± 3.53 mg TE/g, followed by water (336.50 ± 2.19 mg TE/g). Acetone and ethyl acetate showed the weakest activity, with values of 42.53 ± 0.33 and 41.21 ± 0.22 mg TE/g, respectively. A similar trend was observed in the ABTS assay, with the acetone/water extract performing best (510.45 ± 8.60 mg TE/g), followed by water (358.48 ± 11.72 mg TE/g), while ethyl acetate and acetone were the weakest, with values of 117.19 ± 0.11 and 116.26 ± 0.35 mg TE/g, respectively. For *H. lydium*, acetone/water again showed the best quenching ability, followed by the water extract, with values of 297.94 ± 1.27 and 206.48 ± 9.28 mg TE/g in the DPPH assay, and 409.41 ± 13.01 and 267.62 ± 8.26 mg TE/g in the ABTS assay, respectively. The weakest extracts in the DPPH assay were acetone (46.29 ± 0.04 mg TE/g) and ethyl acetate (23.08 ± 0.63 mg TE/g), while in the ABTS assay, acetone and ethyl acetate had values of 113.86 ± 1.19 and 37.87 ± 3.35 mg TE/g, respectively. Regarding reducing capacity for *H. empetrifolium*, the CUPRAC assay showed the best results for the water extract (500.00 ± 5.37 mg TE/g), followed by acetone/water (493.32 ± 6.27 mg TE/g). Acetone and ethyl acetate extracts displayed lower values of 152.35 ± 5.09 and 115.83 ± 7.61 mg TE/g, respectively. In the FRAP assay, the water extract again performed best (297.06 ± 3.49 mg TE/g), followed by acetone/water (275.59 ± 4.38 mg TE/g). Acetone (63.37 ± 0.64 mg TE/g) and ethyl acetate (46.42 ± 5.28 mg TE/g) showed the lowest reducing capacities. For *H. lydium*, the reducing power was lower than that of *H. empetrifolium*, but the order of effectiveness remained the same (water>acetone/water>acetone>ethyl acetate) in both the CUPRAC and FRAP assays. Specifically, in the CUPRAC assay, the water extract showed a value of 303.17 ± 5.59 mg TE/g, followed by acetone/water (266.60 ± 4.91 mg TE/g), acetone (159.31 ± 2.89 mg TE/g), and ethyl acetate (70.10 ± 2.17 mg TE/g). In the FRAP assay, the values were 176.19 ± 1.28 mg TE/g for water, 141.83 ± 2.23 mg TE/g for acetone/water, 75.53 ± 2.74 mg TE/g for acetone, and 24.15 ± 0.48 mg TE/g for ethyl acetate. The phosphomolybdenum assay has attracted attention in recent research due to its simplicity and the lack of requirement for specialized instruments. This method involves the reduction of Mo (VI) to Mo (V) by antioxidant compounds under acidic conditions. In the extracts obtained from *H. empetrifolium*, the highest capacity was observed in the acetone/water extract, with a value of 2.70 ± 0.05 mmol TE/g, followed by water (2.55 ± 0.08 mmol TE/g), ethyl acetate (2.54 ± 0.16 mmol TE/g), and acetone (2.50 ± 0.15 mmol TE/g). Different results were obtained from the *H. lydium* extracts. Although acetone/water showed the highest value again, at 2.87 ± 0.10 mmol TE/g, the second‐best extract in the phosphomolybdenum assay for this species was acetone, with 2.13 ± 0.11 mmol TE/g. The weakest extracts were water and ethyl acetate, with values of 1.92 ± 0.03 and 1.79 ± 0.06 mmol TE/g, respectively. One of the most important antioxidant mechanisms is the chelation of transition metals, which prevents the formation of hydroxyl radicals during the Fenton reaction, thereby helping to reduce oxidative stress. The strongest chelating ability for *H. empetrifolium* was observed in acetone (38.28 ± 1.88 mg EDTAE/g) and ethyl acetate (35.82 ± 6.17 mg EDTAE/g), whereas the weakest were water (17.55 ± 1.35 mg EDTAE/g) and acetone/water (16.12 ± 2.66 mg EDTAE/g). In contrast, for *H. lydium*, the water extract exhibited the highest chelating capacity (40.66 ± 0.40 mg EDTAE/g), followed by ethyl acetate (31.64 ± 1.12 mg EDTAE/g), acetone (27.08 ± 1.08 mg EDTAE/g), and acetone/water (16.15 ± 2.49 mg EDTAE/g). In this study, the antioxidant results showed a correlation with the Folin–Ciocalteu assay for total phenol and flavonoid content, which play an important role in antioxidant activities. For example, the best antioxidant results in both *H. empetrifolium* and *H. lydium* were obtained with acetone/water and water extracts, which were also the richest in TPC and TFC. In the study by Boga et al. (2021), the antioxidant potential of ethanol extracts from the aerial part (HEA) and roots (HER) of *H. empetrifolium* was investigated using the DPPH radical scavenging assay, the ABTS cation radical assay, and the CUPRAC assay. In all antioxidant assays, the HEA extract showed a significant antioxidant effect, with an IC50 value of 11.98 ± 0.22 μg/mL for the DPPH assay, higher than two standard compounds, and 5.32 ± 0.02 μg/mL for the ABTS assay. The antioxidant capacity of *H. lydium* ethanolic extract was evaluated through the DPPH assay by Boran and Ugur (2017). The results showed an IC50 value of 0.165 ± 0.23 mg/mL, higher than BHT, a standard compound, with an IC50 value of 0.184 ± 0.01 mg/mL. However, these results are not directly comparable to ours since, in our study, the results were expressed as Trolox equivalents. In our previous study on two *Hypericum* species (*H. neurocalycinum* and *H. triquetrifolium*), we found that the antioxidant properties of the species were altered by plant parts (aerial parts and roots) and extraction solvents (methanol and water) (Dall'Acqua et al. 2021). For example, in the study, the best DPPH radical scavenging ability was found in the water extract of roots of *H. triquetrifolium* with 407.35 mg TE/g extract, which was higher than the values reported in the current study. For comparison, in our previous study, the results of CUPRAC and FRAP varied between 223.42 and 694.90 mg TE/g and 118.51 and 434.76 mg TE/g, respectively. However, in the current study, these ranges were 70.10–500 mg TE/g and 24.15–297.06 mg TE/g. However, the current value for the metal chelating ability of water extract of *H. lydium* was higher than those of the previous results for *H. neurocalycinum* and *H. triquetrifolium*. The results of these studies, together with our findings, show that the currently studied species have high antioxidant potential, thereby confirming the medicinal properties of other species of the genus *Hypericum* in addition to *H. perforatum* .

**TABLE 3:** Antioxidant properties of the tested extracts.

Species	Solvents	DPPH (mg TE/g)	ABTS (mg TE/g)	CUPRAC (mg TE/g)	FRAP (mg TE/g)	Chelating (mg EDTAE/g)	PBD (mmol TE/g)
*Hypericum empetrifolium*	EA	41.21 ± 0.22^e^	117.19 ± 0.11^e^	115.83 ± 7.61^e^	46.42 ± 5.28^g^	35.82 ± 6.17^ab^	2.54 ± 0.16^b^
	Acetone	42.53 ± 0.33^e^	116.26 ± 0.35^e^	152.35 ± 5.09^d^	63.37 ± 0.64^f^	38.28 ± 1.88^ab^	2.50 ± 0.15^b^
	Acetone/Water	392.71 ± 3.53^a^	510.45 ± 8.60^a^	493.32 ± 6.27^a^	275.59 ± 4.38^b^	16.12 ± 2.66^d^	2.70 ± 0.05^ab^
	Water	336.50 ± 2.19^b^	358.48 ± 11.72^c^	500.00 ± 5.37^a^	297.06 ± 3.49^a^	17.55 ± 1.35^d^	2.55 ± 0.08^b^
*Hypericum lydium*	EA	23.08 ± 0.63^f^	37.87 ± 3.35^f^	70.10 ± 2.17^f^	24.15 ± 0.48^h^	31.64 ± 1.12^bc^	1.79 ± 0.06^d^
	Acetone	46.29 ± 0.04^e^	113.86 ± 1.19^e^	159.31 ± 2.89^d^	75.53 ± 2.74^e^	27.08 ± 1.08^c^	2.13 ± 0.11^c^
	Acetone/Water	297.94 ± 1.27^c^	409.41 ± 13.01^b^	266.60 ± 4.91^c^	141.83 ± 2.23^d^	16.15 ± 2.49^d^	2.87 ± 0.10^a^
	Water	206.48 ± 9.28^d^	267.62 ± 8.26^d^	303.17 ± 5.59^b^	176.19 ± 1.28^c^	40.66 ± 0.40^a^	1.92 ± 0.03^cd^

*Note:* Values are reported as mean ± SD of three parallel measurements. Different letters indicate significant differences between tested extracts (*p* < 0.05).

Abbreviations: EDTAE, EDTA equivalent; MCA, metal chelating activity; PBD, phosphomolybdenum; TE, trolox equivalent.

**FIGURE 2:**
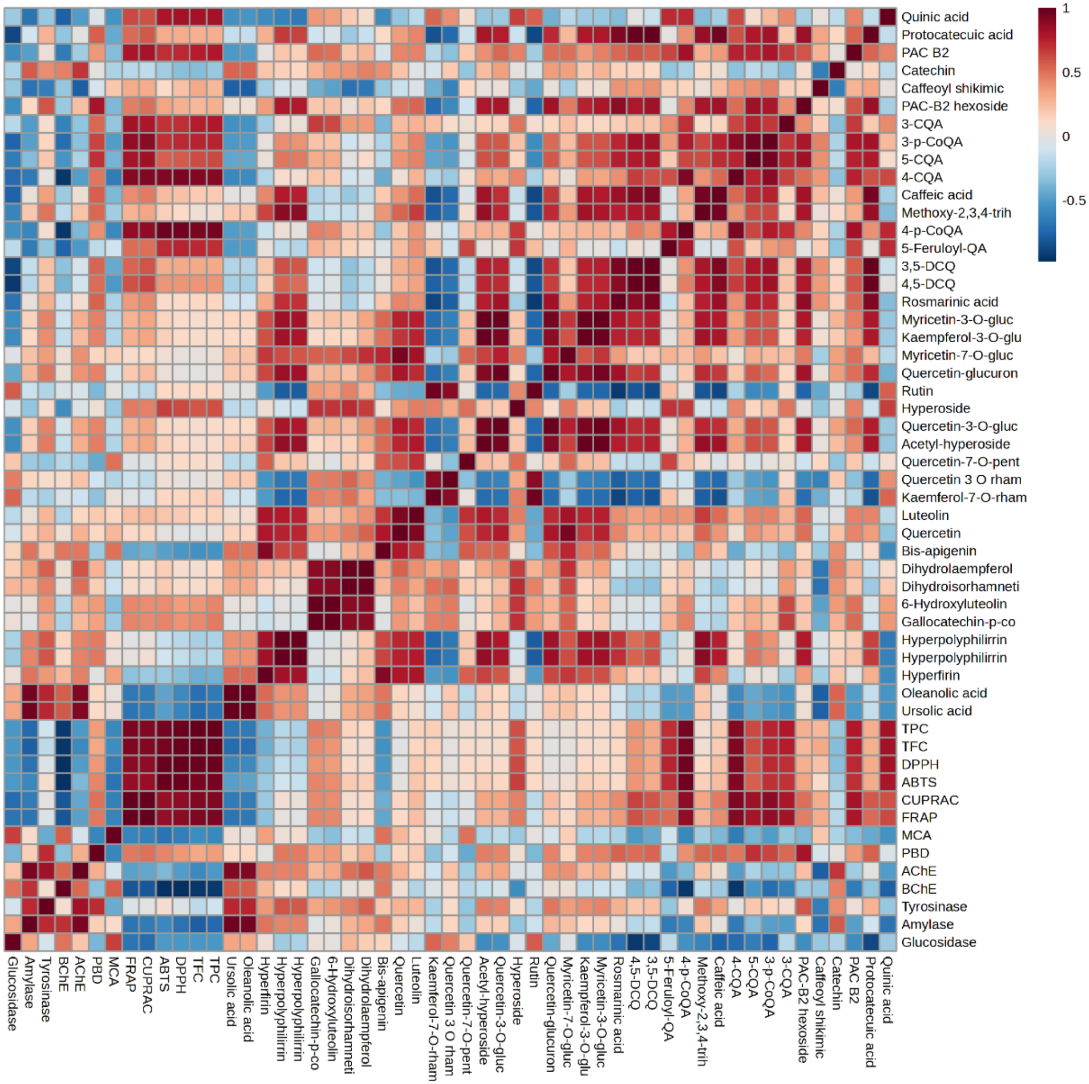
Heatmap obtained from the correlation analysis (Pearson's) of results obtained from chemical characterization of extracts and bioactivity assays (antioxidant and enzyme‐inhibition). Red color indicates a positive correlation and blue color a negative correlation.

The results from chemical characterization of extracts and antioxidant assays were correlated by Pearson's analysis to highlight putative molecular effectors. The results are shown in the heatmap in Figure 2. The highest positive correlation (*p* < 0.001) with radical‐scavenging and metal‐reducing activities was assessed for TPC and TPC, as already expected. Regarding single compounds, the highest positive correlation was assessed for chloroquinic acid derivatives, followed by the two flavonoids hyperoside and 6‐hydroxyluteolin, and gallocatechin‐p‐coumarate (0.01 < *p* < 0.05). A significant inverse correlation was observed with bis‐apigenin and the two triterpenes oleanolic and ursolic acids. Total antioxidant capacity (PDB assay) was positively correlated with PAC‐B2 hexoside, 3‐p‐CoQA, and 5‐CQA (*p* < 0.001), and protocathecuic acid, caffeic acid, methoxy‐2,3,4‐trihydroxycinnamic acid, 3,5‐DCQ and 4,5‐DCQ, and rosmarinic acid (*p* < 0.01). Conversely, a significant (*p* < 0.05) negative correlation was observed with the flavonoid quercetin‐7‐*O*‐pentoside. Correlation with TPC and TFC was positive, although not significant (0.05 < *p* < 0.1). Regarding metal‐chelating activity, the highest correlation (0.01 < *p* < 0.001) was assessed with quercetin‐7‐penstoside and bis‐apigenin, while an inverse correlation was observed with quinic acid, 4‐CQA, and 4‐p‐CoQA (*p* < 0.05).

### 3.4 Enzyme Inhibitory Effects

The growing global impact of non‐communicable diseases (NCDs) calls for the adoption of new therapeutic strategies (WHO 2021). Scientific research increasingly focuses on developing innovative treatments, particularly natural products, due to concerns about the side effects of synthetic compounds (Newman and Cragg 2020). Natural enzyme inhibitors have emerged as promising candidates for managing chronic and degenerative disorders due to their ability to modulate the activity of enzymes involved in the pathogenesis of NCDs (Salehi et al. 2019). Consequently, there is growing interest in screening natural compounds for their ability to inhibit key enzymes such as lipase, α‐amylase, and cholinesterase, which are associated with the development of these diseases (Rigano et al. 2017). In this research, various extracts from *H. empetrifolium* and *H. lydium* were evaluated for their inhibitory effects on several enzymes: cholinesterase (linked to Alzheimer's disease), tyrosinase (involved in Parkinson diseases and also related to skin issues as hyperpigmentation), and both amylase and glucosidase (associated with type‐2 diabetes).

As reported in Table 4, in the AChE inhibition assay, the acetone extract of *H. empetrifolium* showed the highest activity (3.20 ± 0.01 mg GALAE/g), followed by the acetone/water extract (2.96 ± 0.01 mg GALAE/g). Ethyl acetate and water extracts exhibited values of 2.65 ± 0.51 mg GALAE/g and 1.03 ± 0.16 mg GALAE/g, respectively. For *H. lydium*, the acetone extract was also the most potent (2.81 ± 0.11 mg GALAE/g), with the acetone/water extract showing similar results (2.80 ± 0.04 mg GALAE/g). Ethyl acetate demonstrated a value of 2.48 ± 0.10 mg GALAE/g, and water was the weakest (0.89 ± 0.08 mg GALAE/g). Regarding BChE inhibition, the acetone extract of *H. lydium* showed the strongest activity (2.81 ± 0.51 mg GALAE/g), surpassing all *H. empetrifolium* extracts. The ethyl acetate extracts of both species showed moderate BChE inhibition, with *H. lydium* (1.48 ± 0.11 mg GALAE/g) slightly outperforming *H. empetrifolium* (1.36 ± 0.20 mg GALAE/g). Notably, the acetone/water and water extracts of both species were inactive against BChE. Thus, acetone extract of *H. empetrifolium* can be a good starting point for the development of a nutraceutical targeted on AChE and BChE allowing a relative effect of nearly 3 mg Galanthamine per gram of extract, also the extracted components are relatively apolar, and further studies are needed to establish the penetration of the phytochemicals in the SNC.

**TABLE 4:** Enzyme inhibitory properties of the tested extracts.

Species	Solvents	AChE (mg GALAE/g)	BChE (mg GALAE/g)	Tyrosinase (mg KAE/g)	Amylase (mmol ACAE/g)	Glucosidase (mmol ACAE/g)
*Hypericum empetrifolium*	EA	2.65 ± 0.51^ab^	1.36 ± 0.20^b^	55.29 ± 0.28^b^	1.14 ± 0.04^a^	0.83 ± 0.01^a^
	Acetone	3.20 ± 0.01^a^	2.23 ± 0.38^a^	51.93 ± 0.68^c^	1.01 ± 0.01^b^	0.72 ± 0.02^b^
	Acetone/Water	2.96 ± 0.01^ab^	na	66.19 ± 0.30^a^	0.63 ± 0.02^d^	na
	Water	1.03 ± 0.16^c^	na	31.91 ± 0.48^f^	0.17 ± 0.04^e^	na
*Hypericum lydium*	EA	2.48 ± 0.10^b^	1.48 ± 0.11^b^	37.01 ± 1.32^e^	0.85 ± 0.02^c^	0.73 ± 0.02^b^
	Acetone	2.81 ± 0.11^ab^	2.81 ± 0.51^a^	41.26 ± 1.47^d^	0.82 ± 0.02^c^	0.71 ± 0.03^b^
	Acetone/Water	2.80 ± 0.04^ab^	na	66.42 ± 0.64^a^	0.64 ± 0.02^d^	0.84 ± 0.01^a^
	Water	0.89 ± 0.08^c^	na	24.36 ± 0.56 g	0.13 ± 0.01^e^	0.86 ± 0.01^a^

*Note:* Values are reported as mean ± SD of three parallel measurements. Different letters indicate significant differences between tested extracts (*p* < 0.05).

Abbreviations: ACAE: acarbose equivalent; GALAE, galantamine equivalent; KAE, Kojic acid equivalent; na, not active.

Tyrosinase inhibition can be relevant in Parkinson disease and also for the treatment of some skin‐related pathologies. The acetone/water extracts of both species exhibited the highest activity, with *H. lydium* showing 66.42 ± 0.64 mg KAE/g and *H. empetrifolium* showing 66.19 ± 0.30 mg KAE/g. However, *H. empetrifolium* ethyl acetate extract (55.29 ± 0.28 mg KAE/g) showed considerably higher activity compared to the corresponding *H. lydium* extract (37.01 ± 1.32 mg KAE/g).

As reported in a recent review, BchE can be considered as a new strategy for the treatment of various pathologies such as Alzheimer's disease (AD), multiple sclerosis, but also cocaine addiction and detoxifying organophosphorus poisoning, and may play a role in the progression of some of these diseases. New studies have also discovered a possible link between BChE and lipid metabolism, supporting a new potential area of development for the inhibitors. Many data showed that BChE is abnormally increased as a compensator for AChE in the middle and late stages of AD and that the BChE inhibitors may be useful in the remission of cognitive disorders and reduce some pathological features in AD model animals (Qi et al. 2024).

In the amylase inhibition assay, *H. empetrifolium* extracts generally showed higher activity compared to *H. lydium*. The ethyl acetate extract of *H. empetrifolium* demonstrated the strongest inhibition (1.14 ± 0.04 mmol ACAE/g), followed by its acetone extract (1.01 ± 0.01 mmol ACAE/g), and then acetone/water and acetone (0.63 ± 0.02 and 0.17 ± 0.04 mmol ACAE/g, respectively). For *H. lydium*, the ethyl acetate and acetone extracts showed similar but lower inhibition (0.85 ± 0.02 and 0.82 ± 0.02 mmol ACAE/g, respectively). The acetone/water and water extracts showed values of 0.64 ± 0.02 and 0.13 ± 0.01 mmol ACAE/g, respectively. Glucosidase inhibition results varied between the two species. *H. lydium* water extract showed the highest activity (0.86 ± 0.01 mmol ACAE/g), closely followed by its acetone/water extract (0.84 ± 0.01 mmol ACAE/g), then by ethyl acetate extract (0.73 ± 0.02 mmol ACAE/g) and acetone (0.71 ± 0.03 mmol ACAE/g). In contrast, *H. empetrifolium* ethyl acetate extract exhibited the strongest glucosidase inhibition among its extracts (0.83 ± 0.01 mmol ACAE/g). Acetone showed 0.72 ± 0.02 mmol ACAE/g. Interestingly, the acetone/water and water extracts of *H. empetrifolium* were inactive against glucosidase, while these same extracts showed notable activity in *H. lydium*. Also, for the sugar level control these species can be considered as a good starting point for the development of nutraceutical and phytochemical can act directly at intestinal level.

These results highlight that *H. empetrifolium* generally showed stronger inhibition against AChE, tyrosinase, and amylase, while *H. lydium* demonstrated superior activity in BChE and glucosidase inhibition for certain extracts. Other studies have investigated the potential of *H. lydium* and *H. empetrifolium* in inhibiting AChE, BChE, tyrosinase, and amylase, with results expressed as percentages of enzyme inhibition compared to reference standards (Boga et al. 2021; Eruygur et al. 2019). In our previous study in which we considered *H. triquetrifolium* and *H. neurocalycinum* (Dall'Acqua et al. 2021), the enzyme inhibitory effects of root and aerial extracts were studied and in general the methanol extracts were more active than water extracts. For example, AChE inhibition ranged from 0.89 to 3.20 mg GALAE/g in the current study but ranged from 0.63 to 2.48 mg GALAE in our previous study. In addition, the amylase inhibition of ethyl acetate (1.14 mmol ACAE/g) and acetone (1.01 mmol ACAE/g) of *H. empetrifolium* in the current study was higher than those of the extracts of *H. neurocalycinum* and *H. triquetrifolium* in our previous study. These findings suggest that different *Hypericum* species and extraction methods could be targeted for specific enzymatic inhibitions, potentially leading to varied applications in treating related disorders.

Also the results from enzyme inhibition assays were correlated with phytochemical data, as shown in the heatmap reported in Figure 2. Regarding the anti‐cholinesterase activity, different correlations were observed between specific compounds and AChE or BChE. For instance, the highest positive correlation (*p* < 0.001) with AChE was assessed for catechin and the triterpenes oleanolic acid and ursolic acid; for BChE, these latter were found highly correlated, followed by bis‐apigenin (*p* < 0.05). Conversely, caffeoyl shikimic acid was the metabolite showing the most significant negative correlation (*p* < 0.001) with AChE, while quinic acid, PAC B2, and several chloroquinic acids with BChE. Overall, these results suggest that oleanolic acid and ursolic acid may be the phytochemical constituents of highest interest for the antidementia properties of *H. empetrifolium* and *H. lydium* from Turkey. However, further studies are needed to confirm this hypothesis.

Regarding amylase and glucosidase, the highest positive (*p* < 0.001) correlation was observed with oleanolic acid and ursolic acid (amylase), and rutin and kaemferol‐7‐*O*‐rhamnoside (glucosidase, *p* < 0.01). Conversely, the chloroquinic acids 3‐CQA, 3‐p‐CoQA, 5‐CQA, and 4‐CQA were negatively (0.001 < *p* < 0.01) correlated with both anti‐amylase and anti‐glucosidase activities.

Oleanolic and ursolic acids showed a strong (*p* < 0.001) positive correlation also with anti‐tyrosinase activity of studied extracts. This result confirms that these two triterpenes deserve further attention and should be investigated in the future to better understand the molecular mechanisms underlying the different bioactivities exerted by *H. empetrifolium* and *H. lydium*. Significant positive correlations (0.001 < *p* < 0.01) were also assessed for PAC‐B2 hexoside, dihydrolaempferol, dihydroisorhamnetin, and the characteristic phloroglucinols hyperpolyphilirrin (and its isomer) and hyperfirin.

### 3.5 Cytotoxic Effect

The cytotoxic effects of *H. empetrifolium* and *H. lydium* extracts were assessed on three cancer cell lines (MCF‐7, A549, and DU‐145) and one normal cell line (HEK‐293), with IC50 values presented in Table 5. The ethyl acetate extracts exhibited the greatest cytotoxicity across all cell lines. The ethyl acetate extract of *H. lydium* demonstrated the lowest IC50 value in MCF‐7 cells (104.9 μg/mL) while also exhibiting considerable cytotoxicity in normal HEK‐293 cells (IC50 = 63.0 μg/mL), suggesting weak selectivity. In contrast, the ethyl acetate extract of *H. empetrifolium* exhibited marginally elevated IC50 values in cancer cells (141.0–154.2 μg/mL) and reduced toxicity towards normal cells (IC50 = 115.3 μg/mL). Acetone extracts exhibited cytotoxicity, especially in MCF‐7 cells with *H. lydium* (111.1 μg/mL). Water and acetone/water extracts demonstrated minimal cytotoxic effects, with IC50 values surpassing 1000 μg/mL for most cancer cell lines, signifying restricted efficacy (Table 5). The cytotoxic activity of methanol extracts from three *Hypericum* species was previously investigated in vitro on MCF‐7 breast cancer cell lines and PC3 prostate cancer using the MTT method. The study's results showed that cytotoxicity was dependent on concentration in the PC3 cell line (Eruygur et al. 2022). This is consistent with previous findings that demonstrated the strong cytotoxic effects of *Hypericum* extracts; however, our study broadened the focus to include various cancer types alongside a normal cell line for comparison.

**TABLE 5:** Cytotoxic effects of the tested extracts on cancer and normal cell lines (IC_50_ [μg/mL]).

Species	Solvents	DU‐145	A549	HEK‐293	MCF‐7
*Hypericum empetrifolium*	EA	154.2	144.2	115.3	141.0
	Acetone	150.9	150.8	121.8	154.5
	Acetone/Water	188.6	1406.0	303.5	1337.6
	Water	346.9	1584.3	294.0	980.6
*Hypericum lydium*	EA	150.7	143.6	63.0	104.9
	Acetone	192.5	155.4	109.9	111.1
	Acetone/Water	509.3	1032.3	204.6	870.8
	Water	661.0	1126.5	208.6	1059.0

**FIGURE 3:**
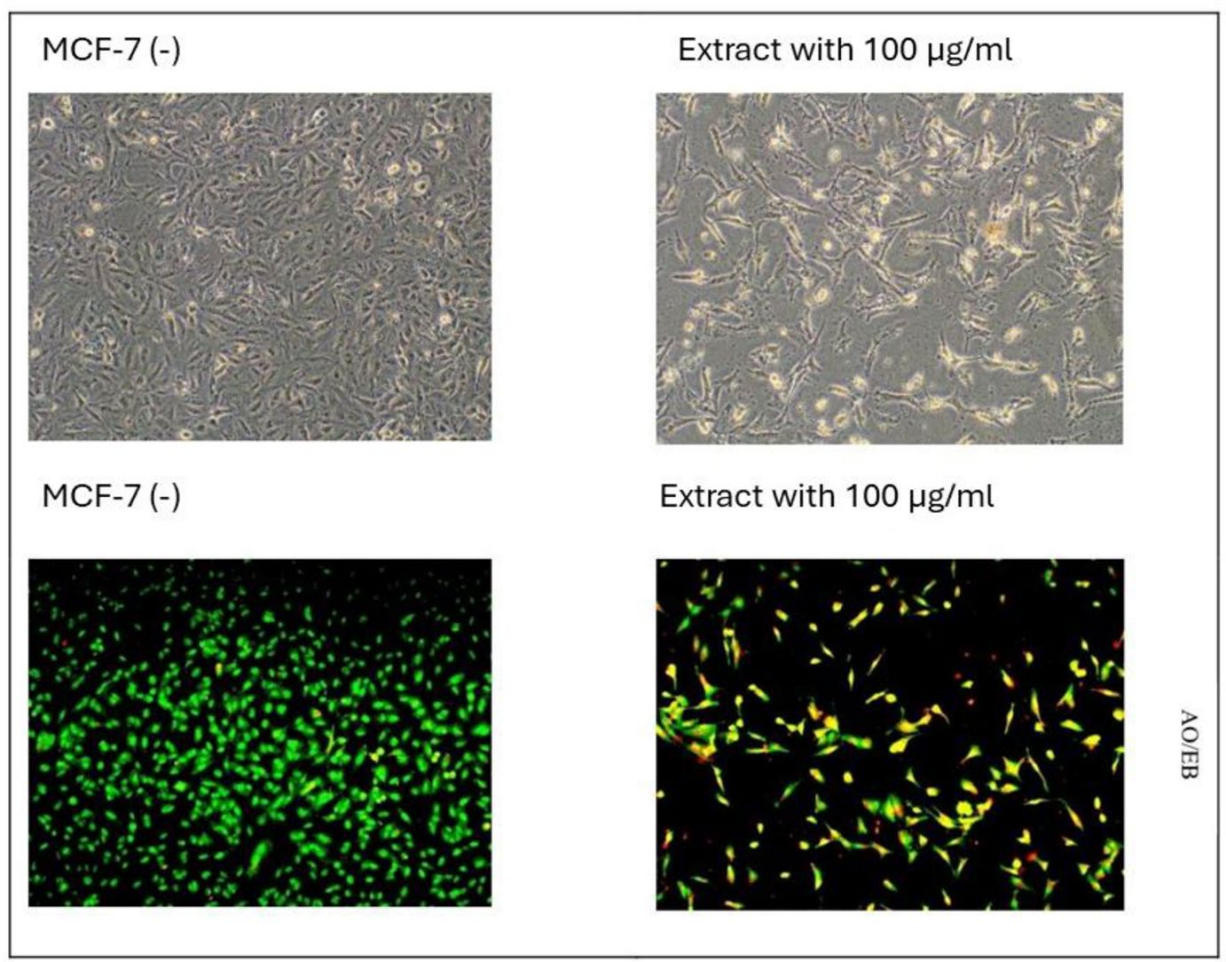
AO/EB staining after the ethyl acetate extract of *Hypericum lydium* (at 100 μg/mL) applied to MCF‐7 cell.

**FIGURE 4:**
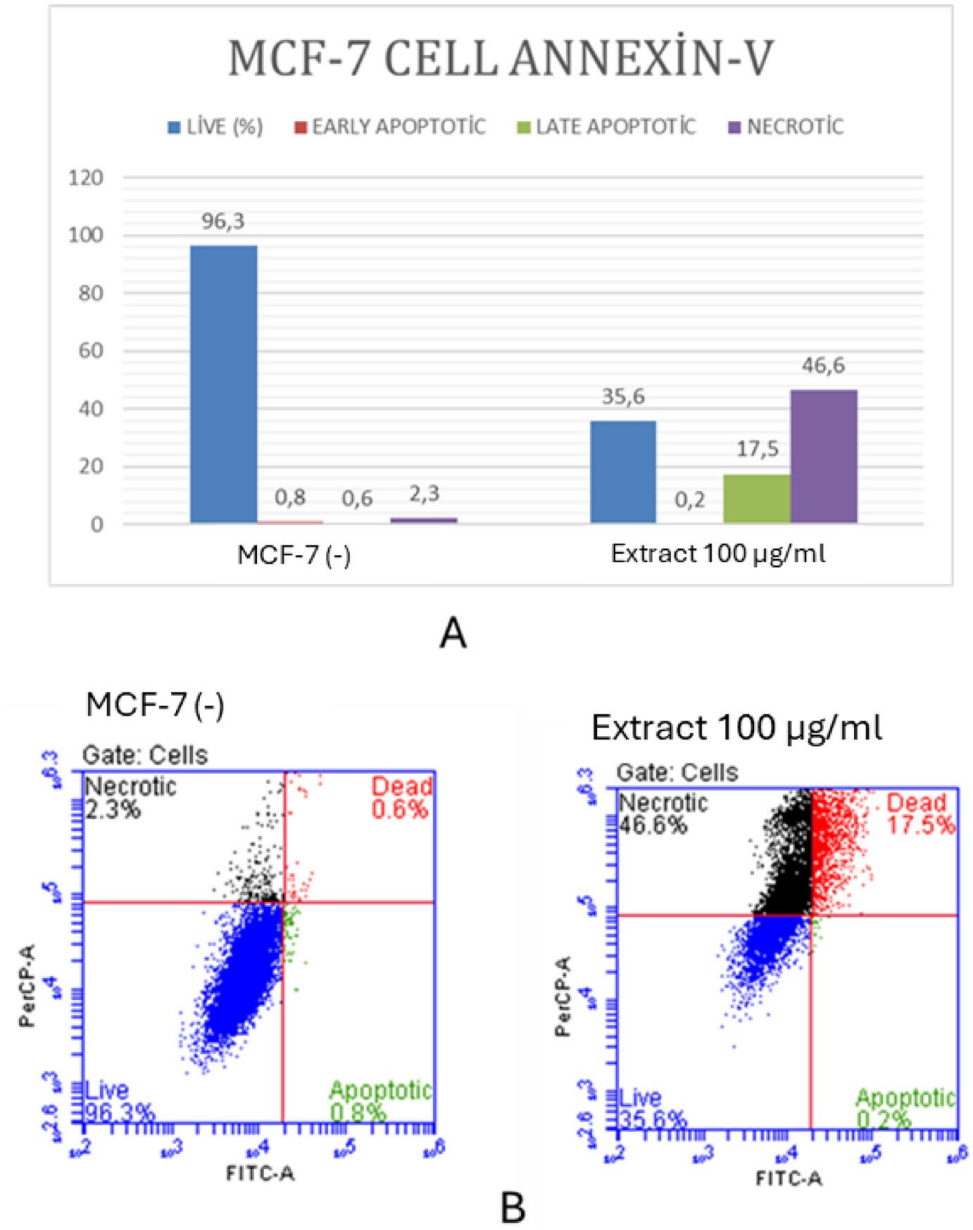
(A) Annexin‐V/PI staining results after the ethyl acetate extract of *Hypericum lydium* (at 100 μg/mL) to MCF‐7 cell. (B) Annexin‐V/PI staining results after the ethyl acetate extract of *Hypericum lydium* (at 100 μg/mL) to MCF‐7 cell. Blue: live‐[(FITC−)/(PI−)]; Green: early apoptotic [(FITC+)/(PI−)]; Red: late apoptotic [(FITC+)/(PI+)]; Black: shows necrotic [(FITC+)/(PI+)] cells.

To further examine the apoptotic effects of *H. lydium* (ethyl acetate extract) on MCF‐7 cells, AO/EB staining, and Annexin‐V/PI tests were conducted. The AO/EB dual staining method using fluorescence‐activated cell sorting (FACS) aims to detect apoptosis‐associated morphological changes in cellular membranes (Gherghi et al. 2003). This approach distinguishes viable, early, late apoptotic, and necrotic cells according to their fluorescence characteristics and morphological features (Baskić et al. 2006). Figure 3 illustrates that the AO/EB staining results demonstrated distinct indicators of apoptosis, such as chromatin condensation and membrane blebbing, accompanied by intense orange fluorescence signifying late‐stage apoptosis (Liu et al. 2015; Prabaharan et al. 2023). This morphological evidence corroborates the cytotoxicity findings obtained from the IC50 testing. The Annexin‐V/PI assay (Figure 4) quantitatively confirmed that the treatment resulted in a significant increase in late apoptotic (17.5%) and necrotic (46.6%) cell populations, in contrast to the control group, which exhibited 96.3% viable cells. The notable increase in apoptotic and necrotic cells corresponds with the decline in cell viability, substantiating that *H. lydium* ethyl acetate extract effectively triggers apoptosis in MCF‐7 cells. Nevertheless, its considerable toxicity to normal HEK‐293 cells indicates restricted selectivity, which may affect its therapeutic efficacy. Additional tuning is necessary to improve selectivity and minimize off‐target effects for prospective cancer therapeutic applications.

#### 3.5.1 Molecular Modeling

**FIGURE 5:**
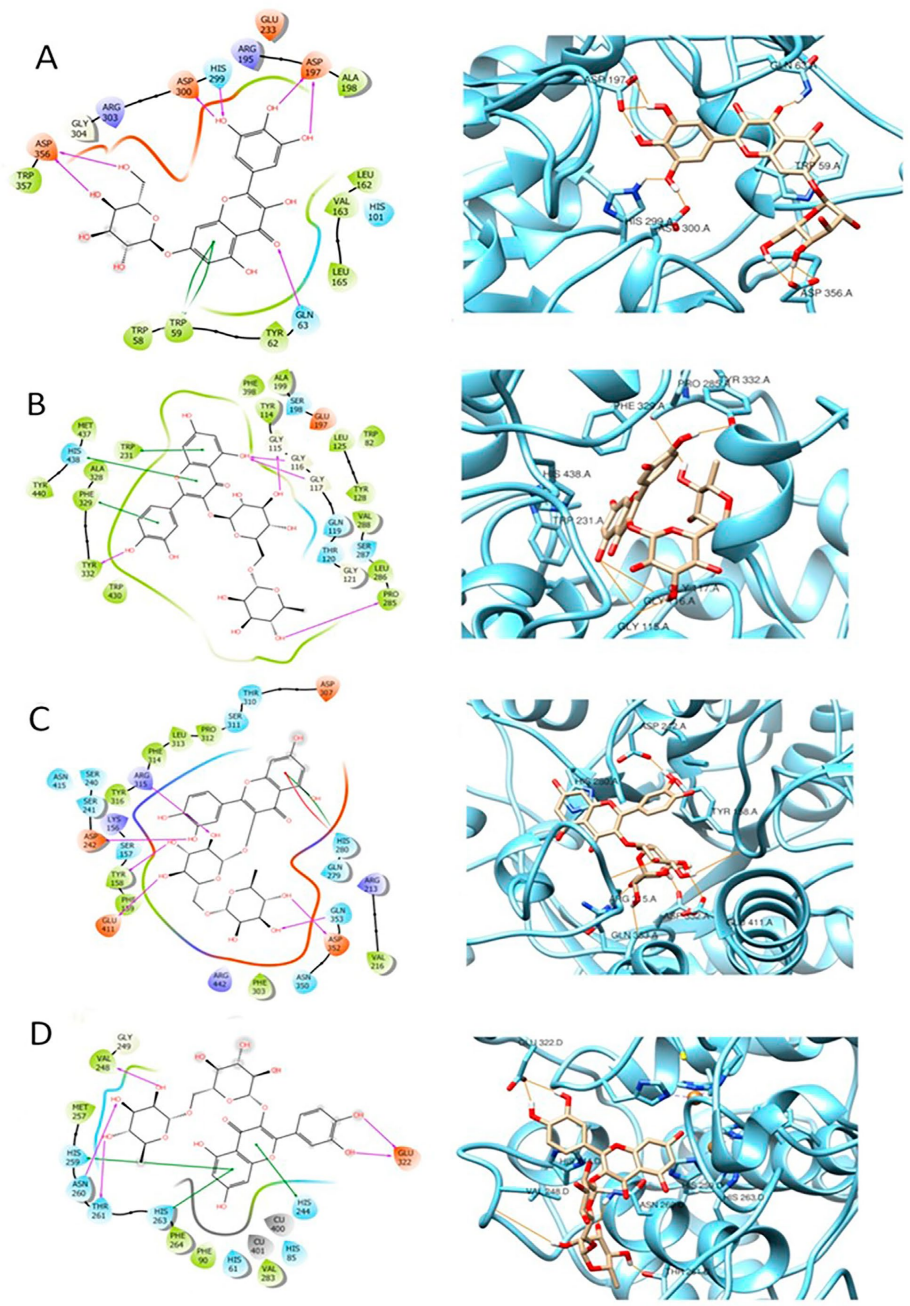
Graphical 2D representation of interactions (left) and binding pose (right) of: **443**‐Amylase (A); **805**‐BChE (B); **805**‐Glucosidase (C); **805**‐Tyrosinase (D).

The best‐ranked docking poses for each protein–ligand system are shown in Table 6. For each protein, the best‐ranked ligand in molecular docking has been chosen to examine interactions, as shown in Figure 5. Compound (**443**) Myricetin‐7‐*O*‐glucoside interacts with Amylase by establishing several H‐bond with Asp356, Asp300, His299, Asp197, Gln63 and a ℼ‐ℼ interaction with Trp59 (Figure 5A). This information is in agreement with previously published article showing the multiple mode of action of myricetin in counteracting diabetes including amylase inhibition, inhibition of glucose transport, agonist activity on Glucagon‐like peptide‐1 receptor in pancreatic cells (Niisato and Marunaka 2023).

**TABLE 6:** Best‐ranking docking poses obtained for each protein–ligand system.

Cps	Tyro SP	Tyro XP	AchE SP	AchE XP	Glucp SP	Gluco XP	But SP	ButXP	Amy SP	Amy XP
805	−6.625	−9.620	−6.540	−12.276	−8.656	−11.263	−9.379	−17.209	−6.721	−10.853
443	−5.994	−7.013	−8.668	−11.228	−7.358	−10.483	−7.835	−14.199	−7.517	−10.948
633	−5.885	−6.213	−7.052	−8.873	−5.831	−7.143	−7.914	−10.607	−4.887	−8.621
343	−5‐531	−4.919	−9.310	−10.718	−7.156	−8.816	−9.035	−10.081	−7.041	−8.458
643	−5.291	−6.871	−8.091	−13.940	−7.797	−8.824	−9.298	−13.761	−8.240	−10.317
856	−5.131	−6.364	—	−6.390	−6.812	−6.653	−9.502	−10.518	−8.058	−8.613
785	−5.118	−6407	−7.116	−8.544	−6260	−6.835	−8.256	−9.598	−5.071	−4.539
915	−5.042	−5.817	−8.144	−10.953	−7.754	−9.512	−9.424	−11.056	−7.421	−8.296
666	−5.097	−6.525	−6.653	−9.265	−6.797	−8.971	−6.780	−9.171	−7.245	−9.175
Rih	−3.732	−4.572	—	−8.720	−4.236	−3.713	−6.236	−7.304	−3.536	−3.436
298	−3.265	−4.592	—	—	−4.362	−3.483	−7.675	−8.190	−2.379	−3.310

Compound (**805**) rutin appears to show the best docking score among all the ligands docked to BChE, glucosidase, and tyrosinase.

When interacting with BChE, compound **805** forms four H‐bond with Tyr332, Gly115, Gly116, Gly117, Pro285 and two main ℼ‐ℼ interactions with Phe329 and Trp231 (Figure 5B). When interacting with Glucosidase, **805** docking pose is stabilized by the formation of several H‐bonds with Glu411, Tyr158, Asp242, Arg315, Gln353, Asp352, a ℼ‐ℼ interaction and a ℼ‐cation interaction, both with His280 (Figure 5C). With Tyrosinase, compound **805** forms H‐bonds with Val248, Asn260, Thr261, Glu322, and ℼ‐ℼ interactions with His259, His263, and His244 (Figure 5D).

**FIGURE 6:**
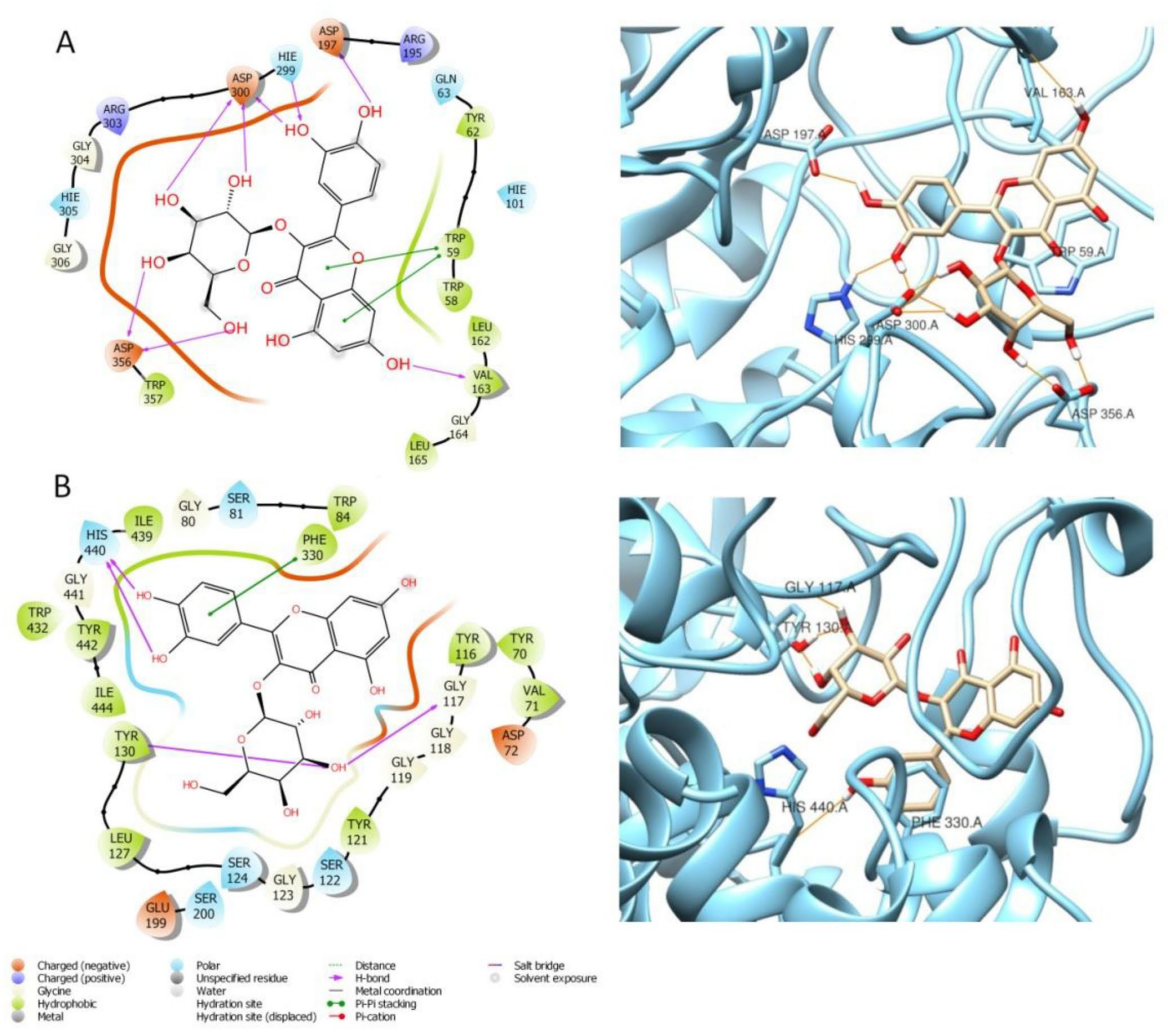
Graphical 2D representation of interactions (left) and binding pose (right) of: **643**‐Amylase (A); **643**‐AChE (B).

Quercetin‐3‐*O*‐galactoside (compound **643**), has been found in high amounts in both species, *H. empetrifolium* and *H. lydium*. Quercetin‐3‐*O*‐galactoside, when interacting with AChE, forms H‐bonds with His440, Gly117, Tyr130, and a ℼ‐ℼ interaction with Phe330 (Figure 5B) and when interacting with Amylase, (docking score: −10.317) Quercetin‐3‐*O*‐galactoside forms an extensive network of H‐bonds with Asp197, His299, Asp300, Asp356, Val163 and shows ℼ‐ℼ interactions with Trp59 (Figure 6A).

On the other hand, when docked to AChE, Quercetin‐3‐*O*‐galactoside exhibits a high XP docking score, equal to −13.940. Therefore, it is rational to think that this compound may have notable activity on both AChE, and Amylase, due to several stable interactions.

**FIGURE 7:**
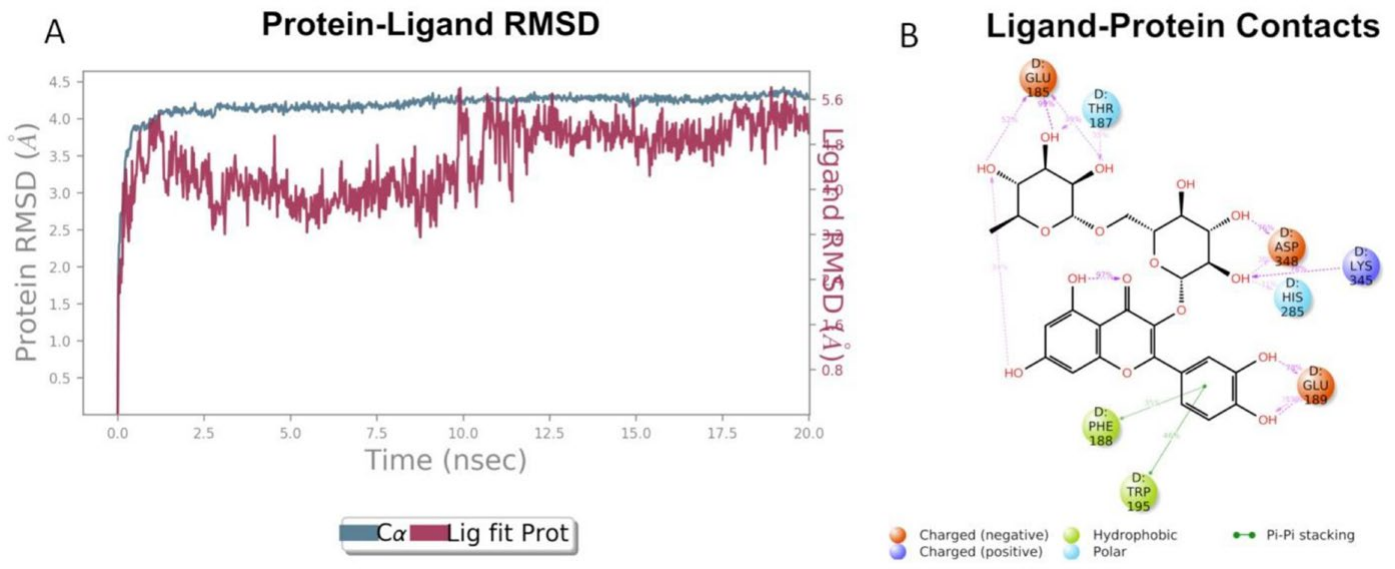
(A) RMSD graphic of Cα of tyrosinase (blue) and compound **805** (red) over a Molecular Dynamic simulation of 20 ns; (B) 2D representation of the interactions between compound **805** and Tyrosinase during the 20 ns Molecular Dynamics experiment.

Since compound **805** shows the best docking score when interacting with Tyrosinase, along with the highest biological activity, this protein–ligand system was selected for molecular dynamics experiments. RMSD and ligand–protein interactions were analyzed by using simulation interaction diagram (SID) panel included in Desmond and are shown in Figure 7.

From the graphic in Figure 7A it is possible to see that the protein remains quite stable at 4.0 Å RMSD for the entire 20 ns experiment, whereas the ligand appears to oscillate around 2.5 to 4.5 Å during the MD experiment. Figure 6B depicts important contacts between Tyrosinase and compound **805**. The most important interactions are maintained over the duration of the experiment, in particular H‐bonds with Glu185 (99%), Lys345 (78%), Glu189 (78%), and Asp348 (76%). It is reasonable to think that these interactions may have a key role in biological activity, since compound **805** appears to have a high affinity for the enzymatic tyrosinase pocket. On the other hand, the docked ligand doesn't reach the catalytic Cu atoms set in the center of the enzymatic pocket; thus it can be speculated that it acts by occlusion of the active site entrance as postulated for other enzymes inhibition mechanism such as carbonic anhydrase [42]. A complete review of the possible role as anticholinesterase inhibitors of flavonoids have been published showing that the maximum AChE inhibitory activity of most of flavonoids is due to the presence and position of hydroxyl (OH) group at ring A and ring B, and due to the unsaturation of ring C, thus suggesting in quercetin derivatives good candidates (Khan et al. 2018).

## 4 Conclusions

The work show the ability of two *Hypericum* species, namely *H. empetrifolium* and *H. lydium* to produce different type of phytochemicals. In terms of chemical composition, we were able to detect specific compounds for the *Hypericum* genus such as hyperoside and hyperfirin in the tested extracts, furthermore differences were observed considering the two species showing that hyperpolyphyllirin, dicaffeoyl quinic acid, and myricetin derivatives are only detected in the *H. empetrifolium* extracts while rutin, quercetin‐3‐*O*‐rhamnoside ant the triterpenoids oleanolic and ursolic acid were only detected in *H. lydium*. To assess potential usefulness in nutraceutical application antioxidant and metal chelating ability of the extracts obtained with different solvents were evaluated. In antioxidant testing, acetone/water and water extracts were generally more active than ethyl acetate and acetone extracts. Furthermore, the ability of the extracts to inhibit some important enzymes have been evaluated namely acetyl, butyril cholinesterase, and tyrosinase all enzymes involved in CNS degenerative diseases. Also alpha and beta glucosidase inhibitory properties were explored. The most lipophilic extracts present significant activities. Also in vitro cell based assays were adopted. The ethyl acetate extract of *H. lydium* showed the strongest cytotoxic effect on the MCF‐7 cell line, but the extract triggered the necrotic process. To gain more insights into the chemical components and the enzymes tested, molecular docking was performed and good interactions between rutin and tyrosinase were observed suggesting a potential role of this flavonoid in the activities involving this enzyme.

In summary, the presented results can provide important information for the development of novel nutraceutical and food supplements based on these plants. Main target that we have demonstrated that can be involved are AChE and BChE, as well as tyrosinase and amylase. Molecular docking studies revealed significant possible role of rutin and of Quercetin‐3‐*O*‐galactoside, rutin for the inhibition of the cholinesterase and tyrosinase, but a possible role of such compounds on SNC diseases point out the need to study the penetration of the compounds in the brain and related tissues. Considering the amylase Myricetin‐7‐*O*‐glucoside was shown from the in silico data as a good candidate. With these results we can indicate the *H empetrifolium* and *H. lydium* as good starting materials for the development of new health promoting agents in the clinical applications including the treatment of diabetes mellitus, Alzhiemer's diseases and pigmentation problems. However, further studies are needed to better understand which compound is responsible for the observed biological activities and how the gut microbiota affects the observed biological activities.

## Author Contributions

Conceptualization, S.S., S.D., G.Z., methodology, S.S., G.Z., G.A.F., G.C., S.S., E.Y., G.Z. software, S.D., İ.K., O.Y., C.E., R.V.; validation, P.A., G.Z., A.M., E.P., L.M.; formal analysis, E.Y., G.Z..; investigation, S.S., S.D., G.Z., G.C., A.M.; resources, S.S., E.Y., G.Z.; data curation G.Z., S.S., S.D., A.M.; writing – original draft preparation, S.S., S.D., G.A.F., G.C., A.M., G.Z.; writing. – review and editing, İ.K., O.Y., C.E., R.V., P.A..; visualization, A.M., E.P., L.M.; supervision, G.Z.; project administration, S.S., S.D., G.Z.; funding acquisition, G.Z. All authors have read and agreed to the published version of the manuscript.

## Ethics Statement

The authors have nothing to report.

## Consent

The authors have nothing to report.

## Conflicts of Interest

The authors declare no conflicts of interest.

## Supporting information


Data S1

## Data Availability

The authors have nothing to report.
