# Supplementary material for: Hypericum empetrifolium and H. lydium as Health Promoting Nutraceuticals: Assessing Their Role Combining In Vitro In Silico and Chemical Approaches
Source: Food Sci Nutr. 2025 Mar 31;13(4):e70053. doi: 10.1002/fsn3.70053 (PMC11957996; doi:10.1002/fsn3.70053)
Supplement: Supplementary file 1 — Data S1 [file FSN3-13-e70053-s001.pptx]

## Slide 1
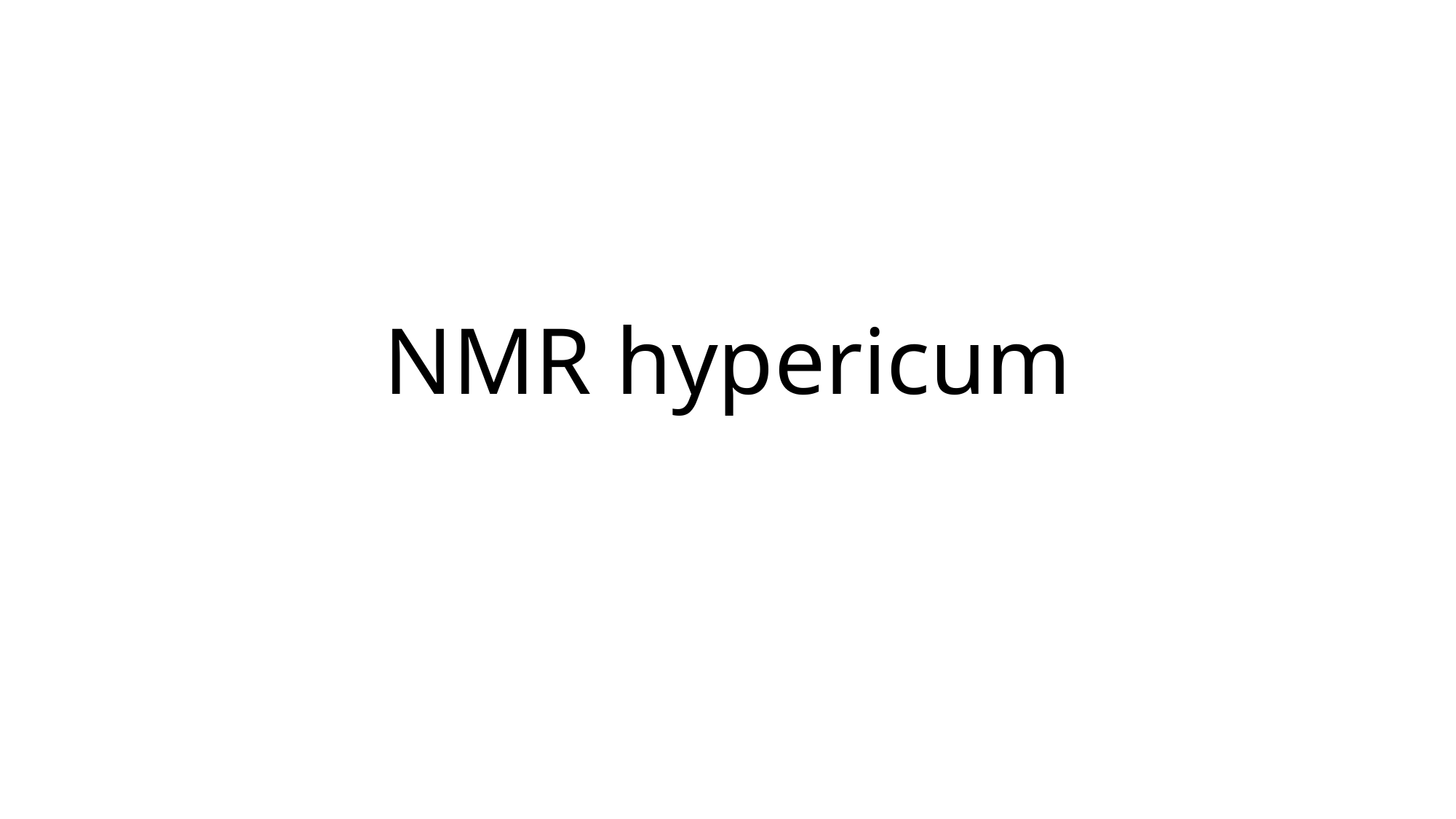

# NMR hypericum

## Slide 2
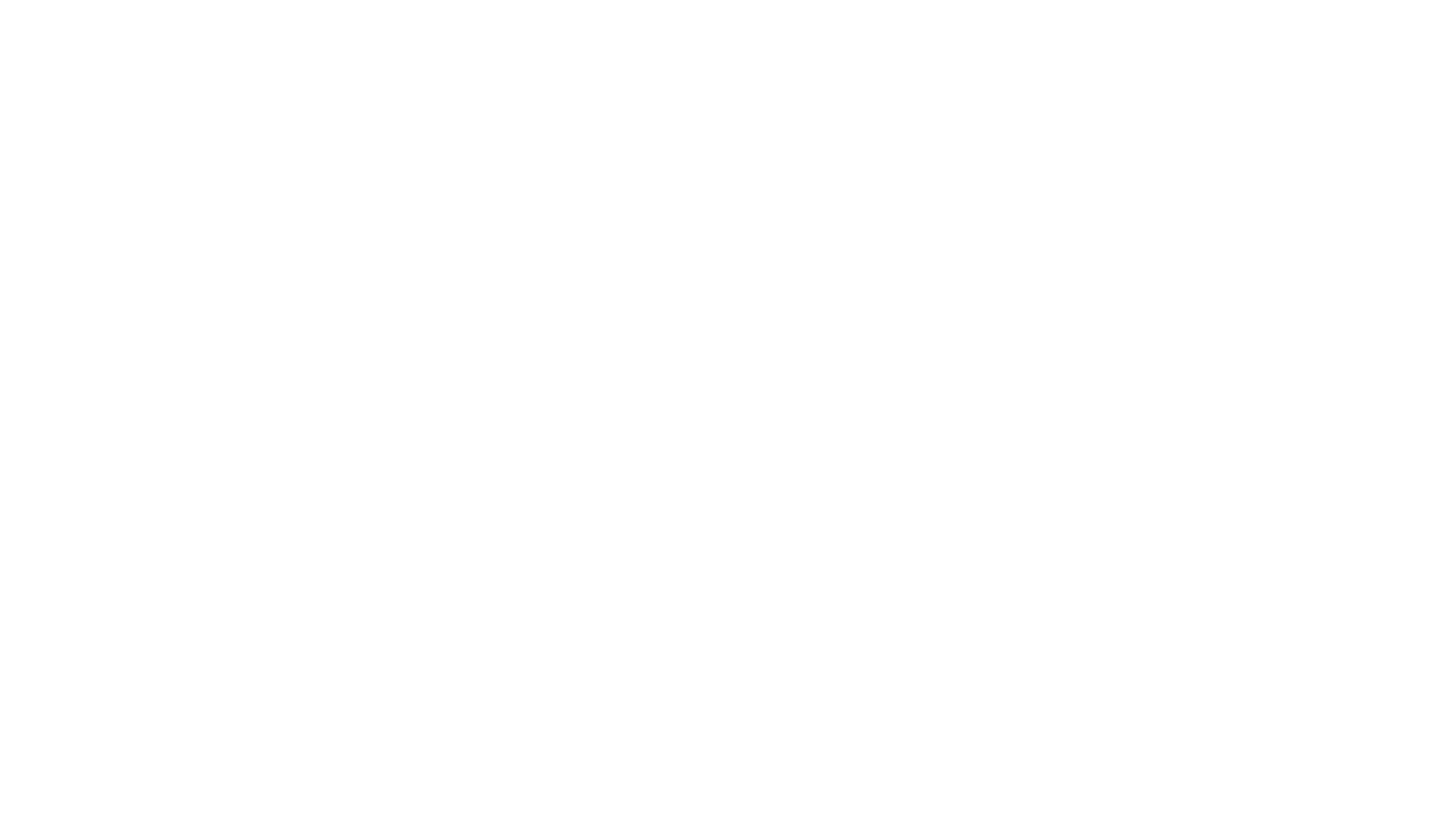

## Slide 3
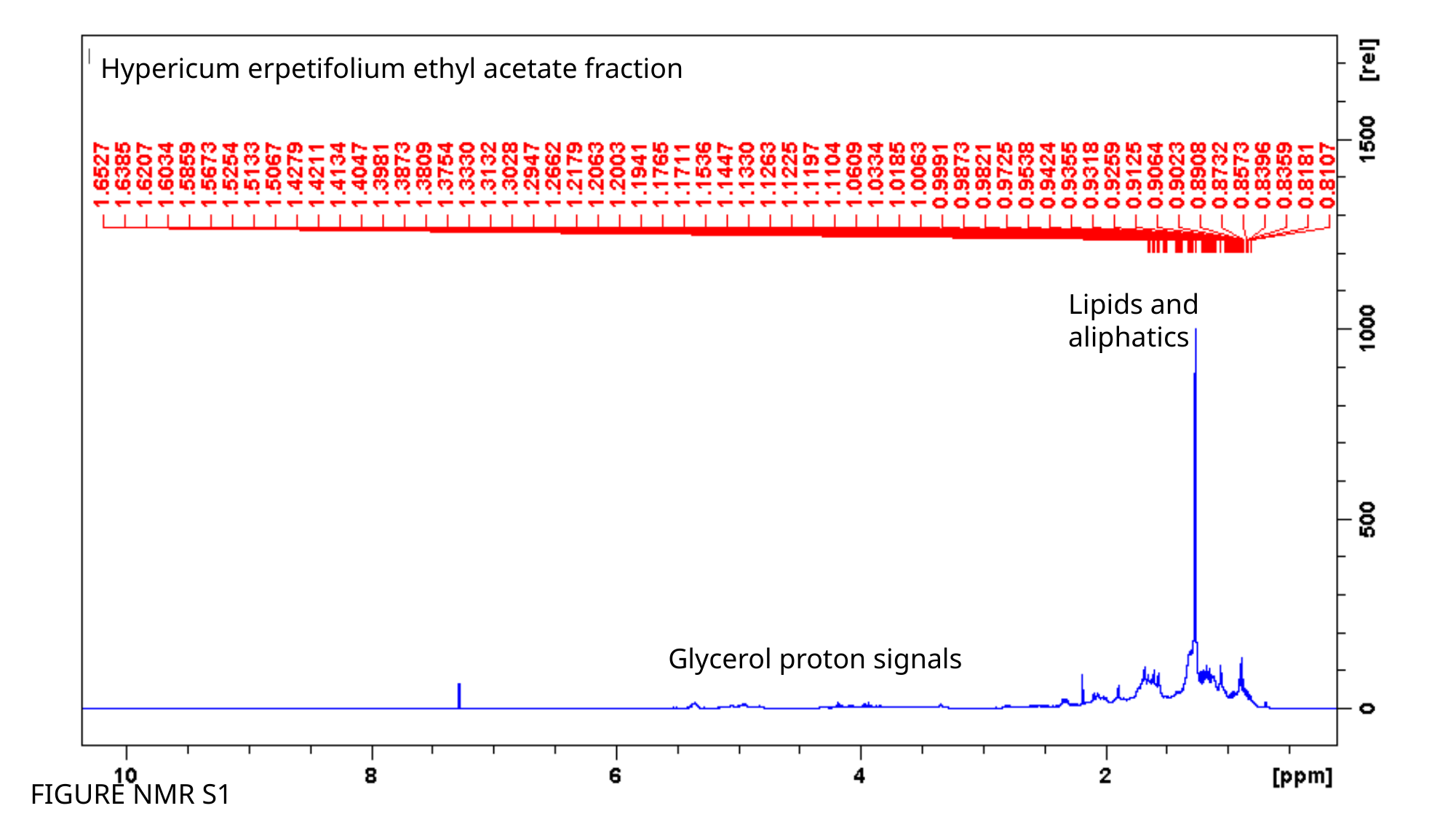

Hypericum erpetifolium ethyl acetate fraction
Lipids and aliphatics
Glycerol proton signals
FIGURE NMR S1

## Slide 4
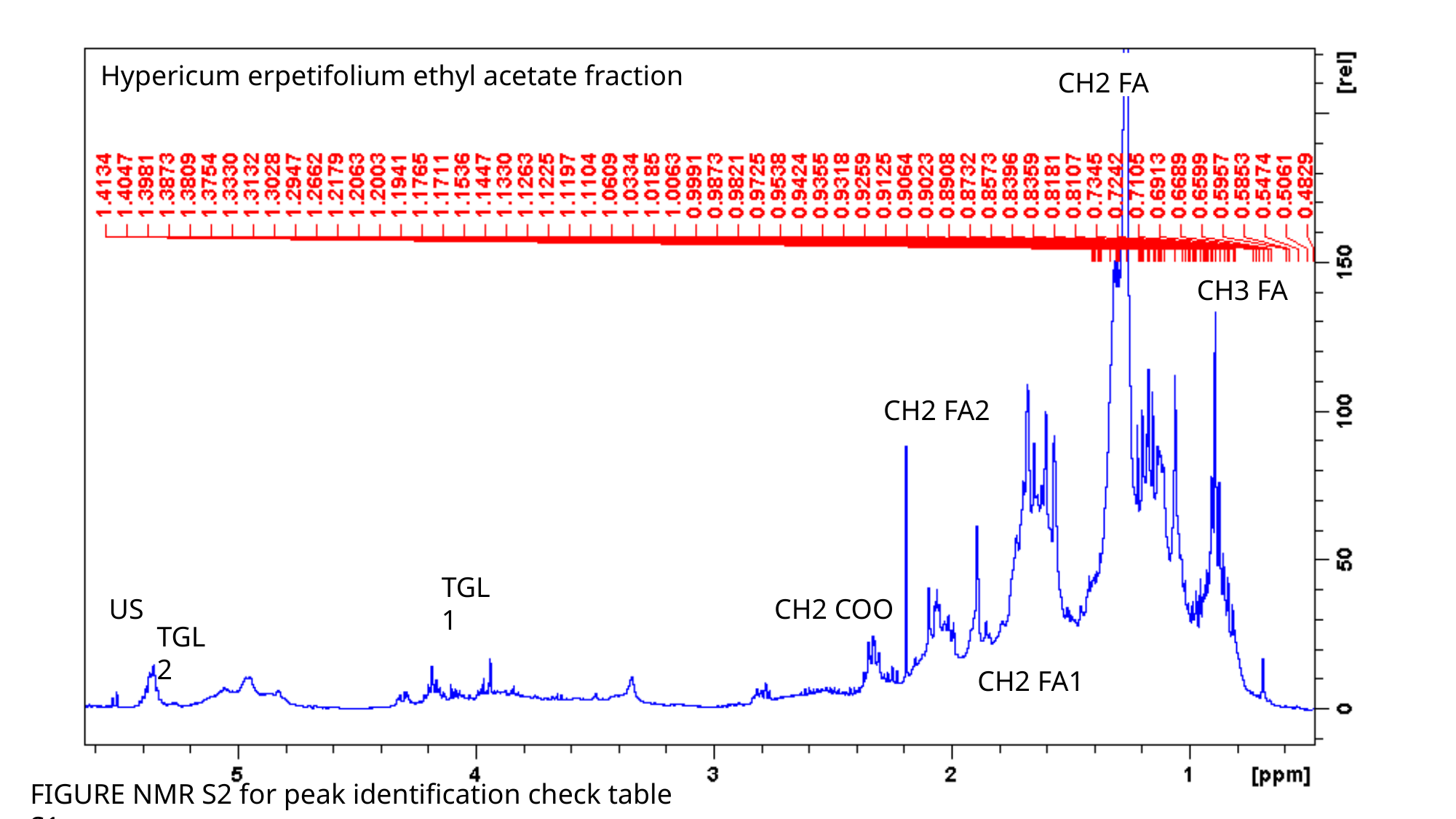

Hypericum erpetifolium ethyl acetate fraction
CH2 FA
CH3 FA
CH2 FA2
TGL1
US
CH2 COO
TGL2
CH2 FA1
FIGURE NMR S2 for peak identification check table S1

## Slide 5
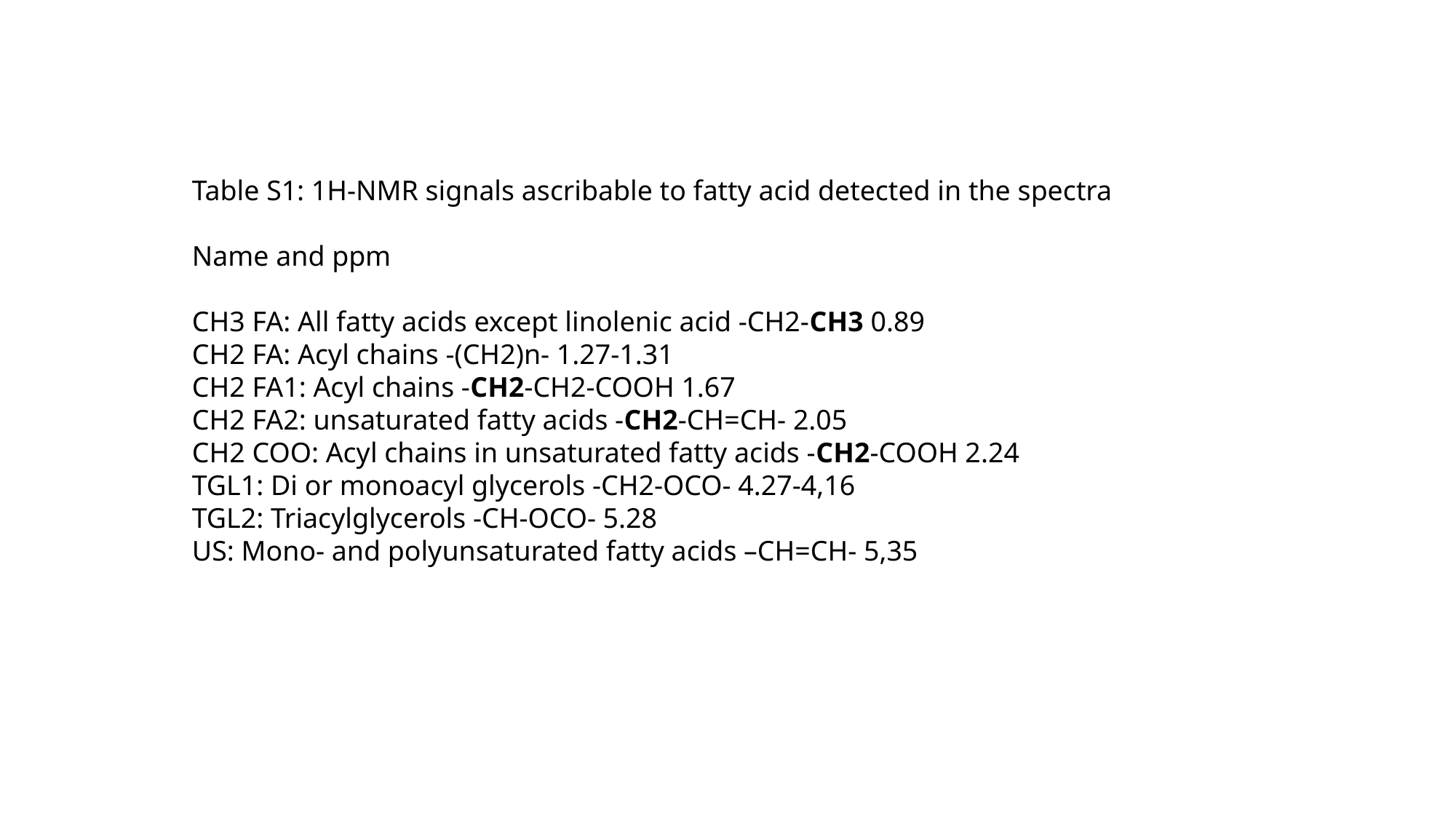

Table S1: 1H-NMR signals ascribable to fatty acid detected in the spectra
Name and ppm
CH3 FA: All fatty acids except linolenic acid -CH2-CH3 0.89
CH2 FA: Acyl chains -(CH2)n- 1.27-1.31
CH2 FA1: Acyl chains -CH2-CH2-COOH 1.67
CH2 FA2: unsaturated fatty acids -CH2-CH=CH- 2.05
CH2 COO: Acyl chains in unsaturated fatty acids -CH2-COOH 2.24
TGL1: Di or monoacyl glycerols -CH2-OCO- 4.27-4,16
TGL2: Triacylglycerols -CH-OCO- 5.28
US: Mono- and polyunsaturated fatty acids –CH=CH- 5,35

## Slide 6
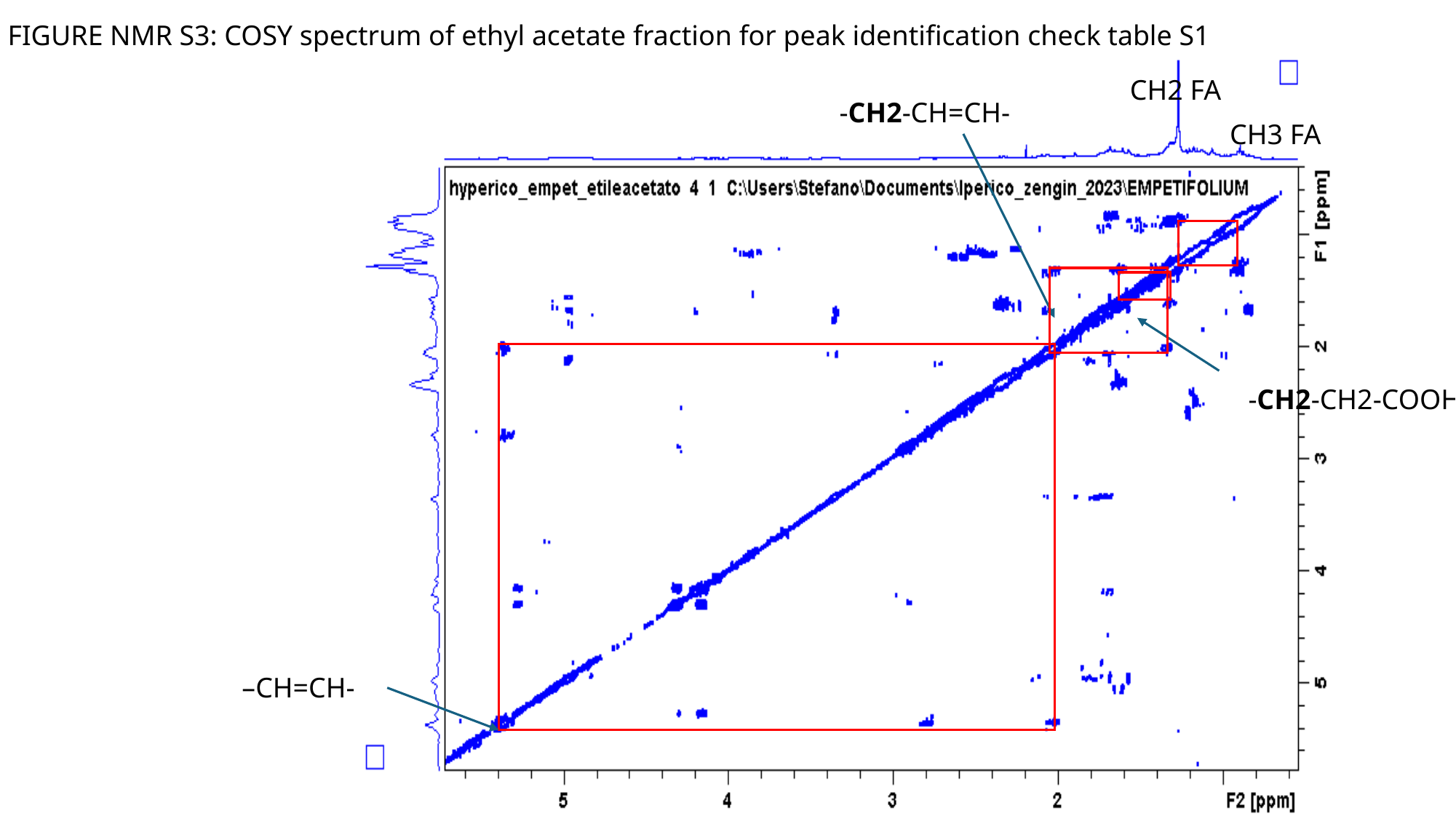

FIGURE NMR S3: COSY spectrum of ethyl acetate fraction for peak identification check table S1
CH2 FA
-CH2-CH=CH-
CH3 FA
-CH2-CH2-COOH
–CH=CH-

## Slide 7
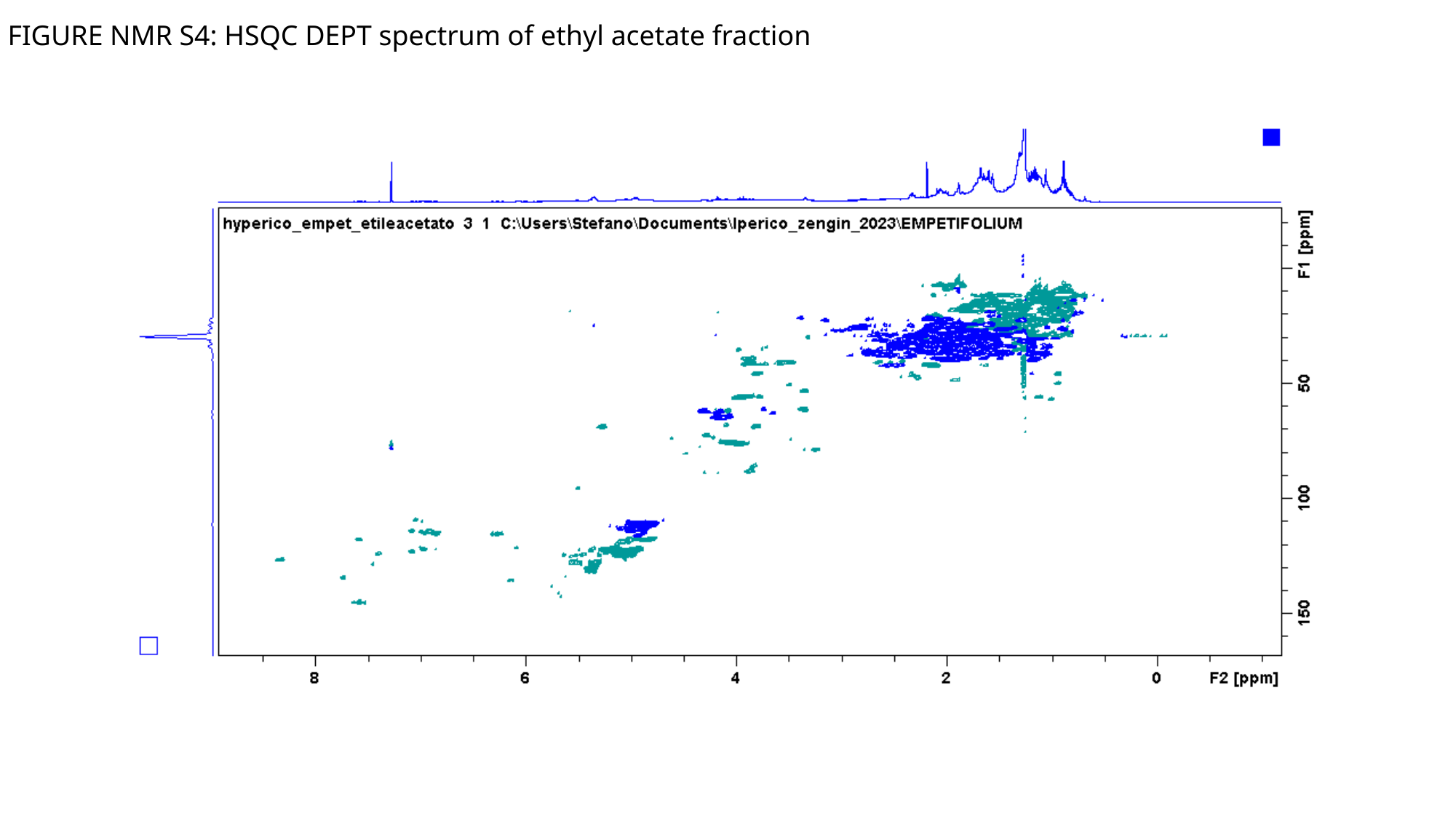

FIGURE NMR S4: HSQC DEPT spectrum of ethyl acetate fraction

## Slide 8
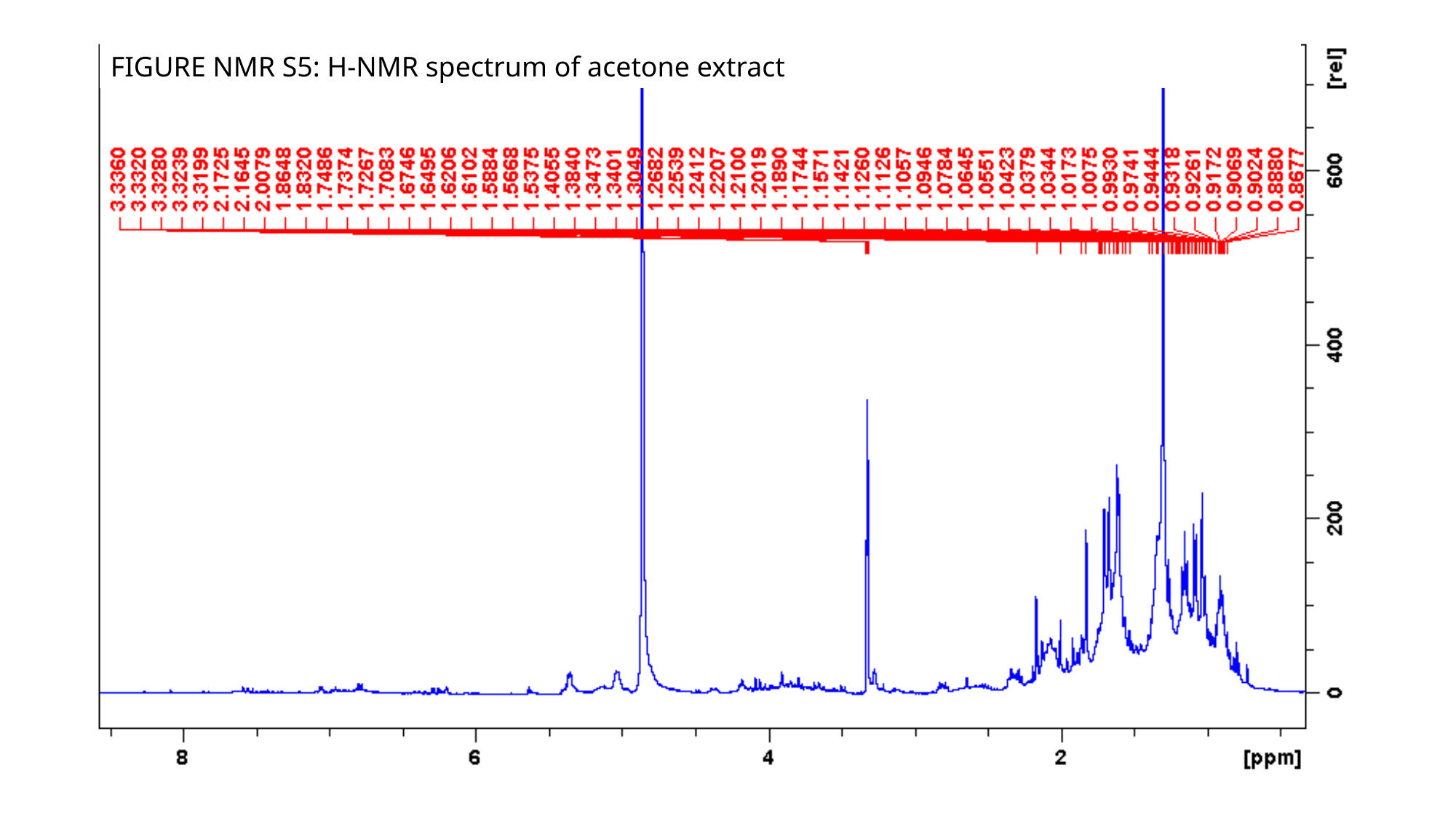

FIGURE NMR S5: H-NMR spectrum of acetone extract

## Slide 9
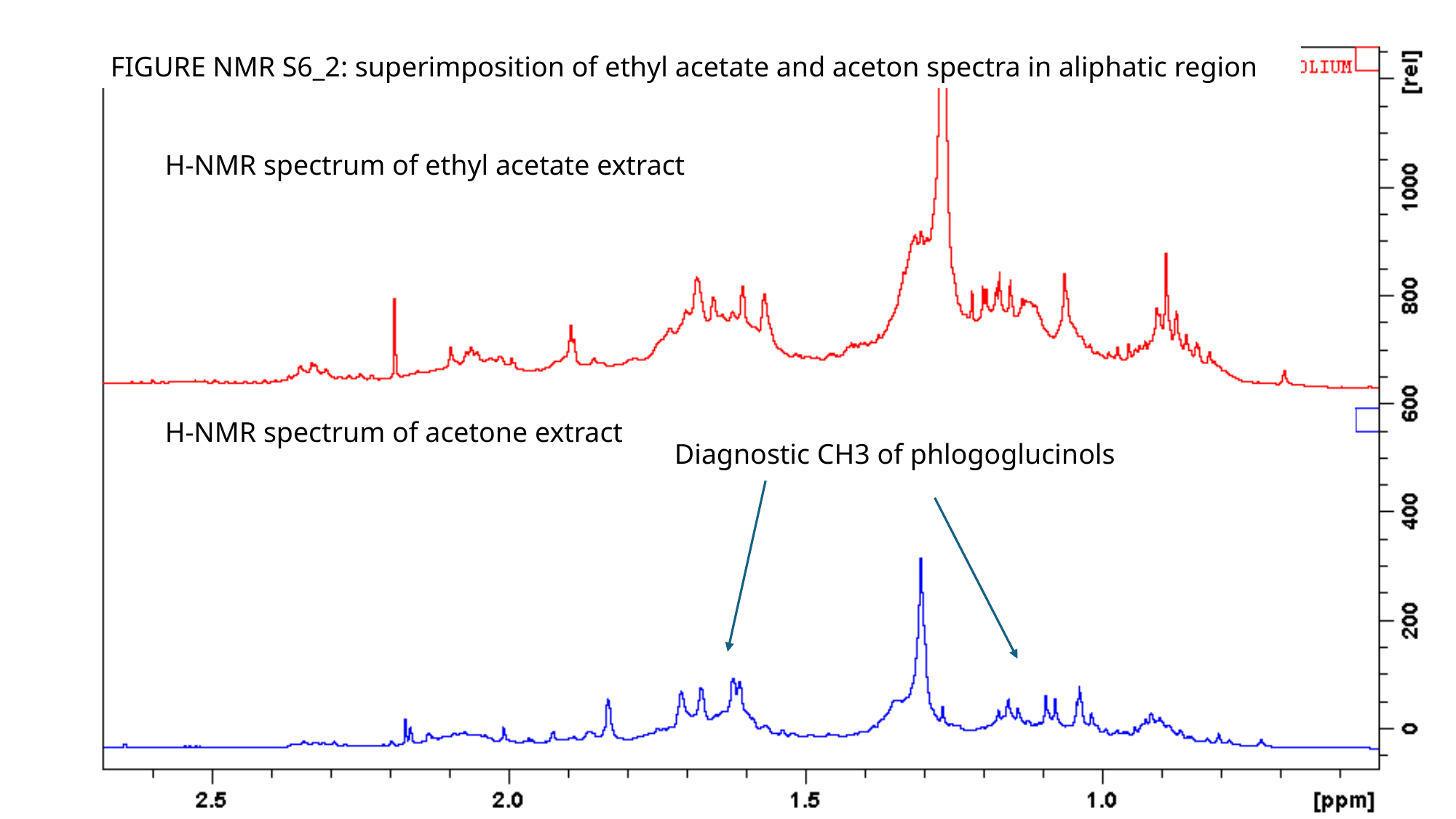

FIGURE NMR S6_2: superimposition of ethyl acetate and aceton spectra in aliphatic region
H-NMR spectrum of ethyl acetate extract
H-NMR spectrum of acetone extract
Diagnostic CH3 of phlogoglucinols

## Slide 10
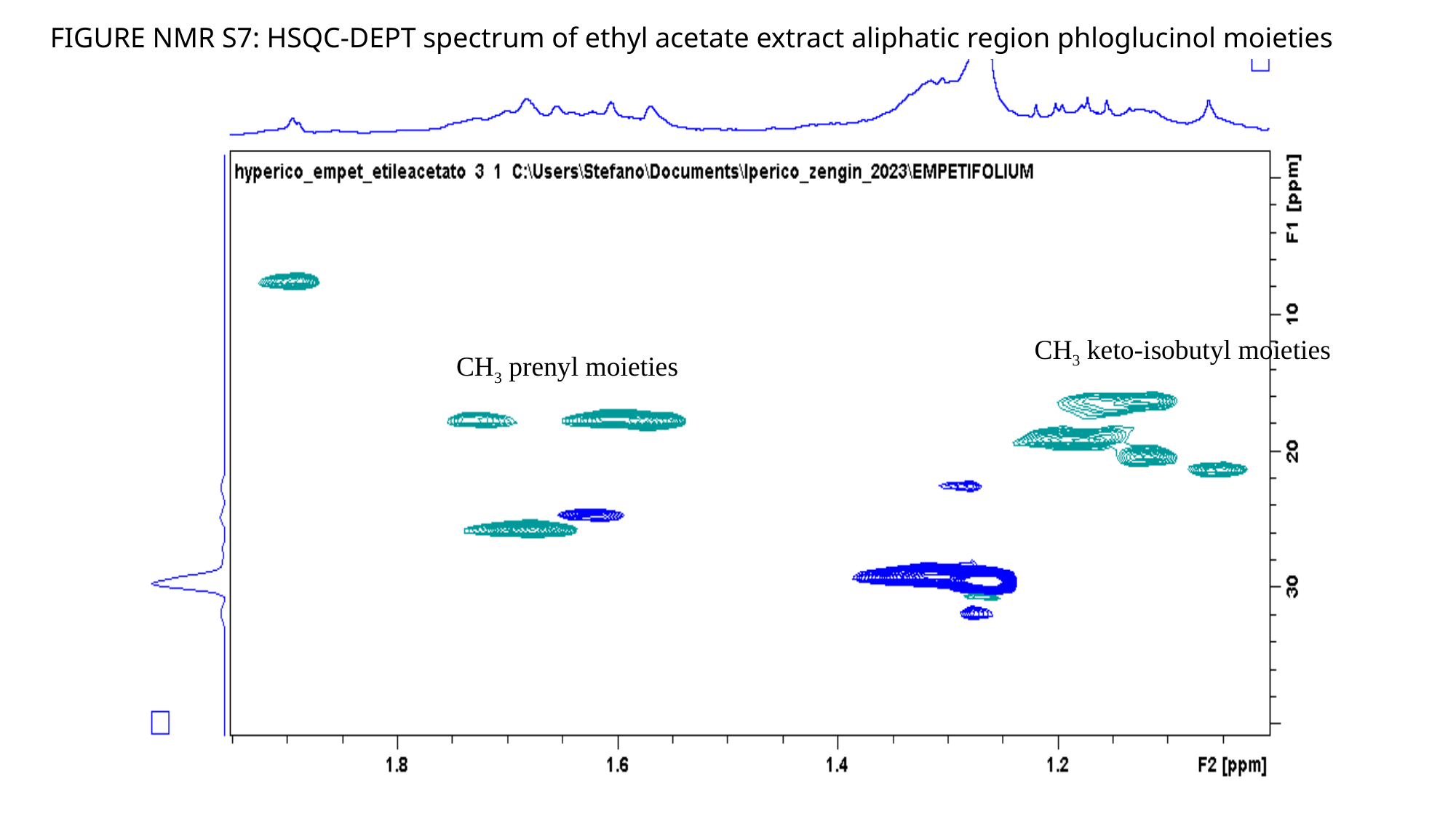

FIGURE NMR S7: HSQC-DEPT spectrum of ethyl acetate extract aliphatic region phloglucinol moieties
CH3 keto-isobutyl moieties
CH3 prenyl moieties

## Slide 11
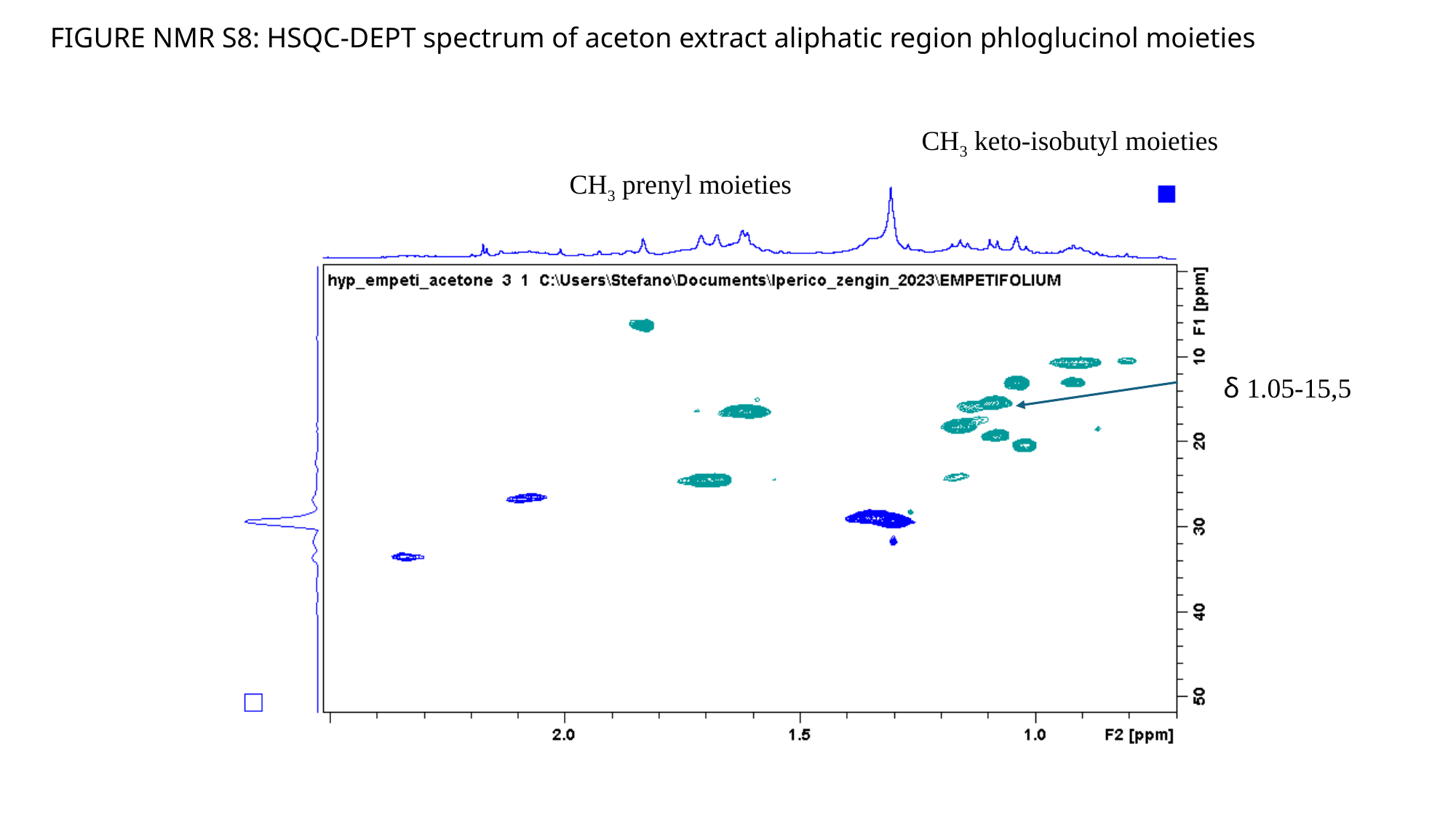

FIGURE NMR S8: HSQC-DEPT spectrum of aceton extract aliphatic region phloglucinol moieties
CH3 keto-isobutyl moieties
CH3 prenyl moieties
δ 1.05-15,5

## Slide 12
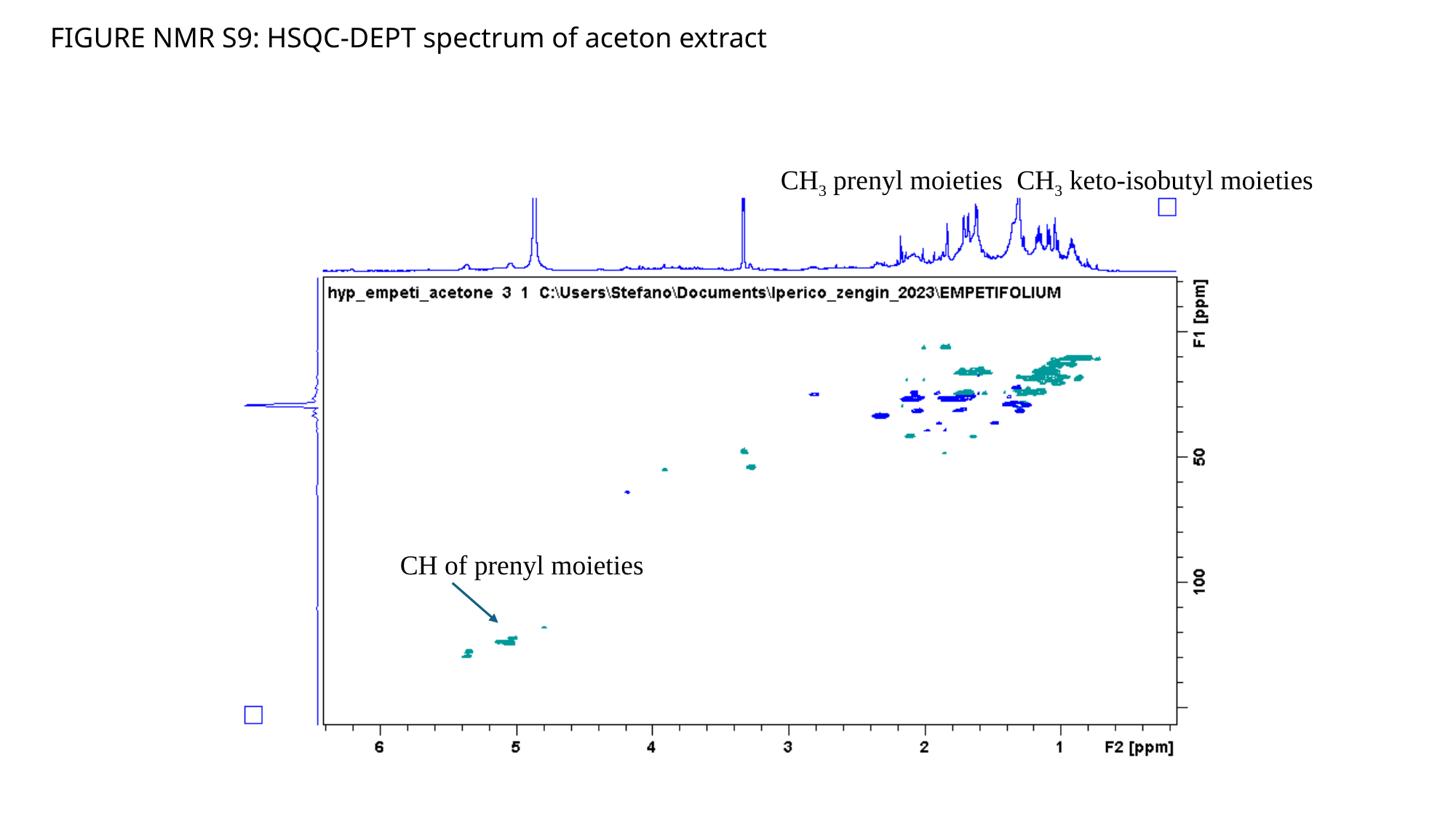

FIGURE NMR S9: HSQC-DEPT spectrum of aceton extract
CH3 prenyl moieties
CH3 keto-isobutyl moieties
CH of prenyl moieties

## Slide 13
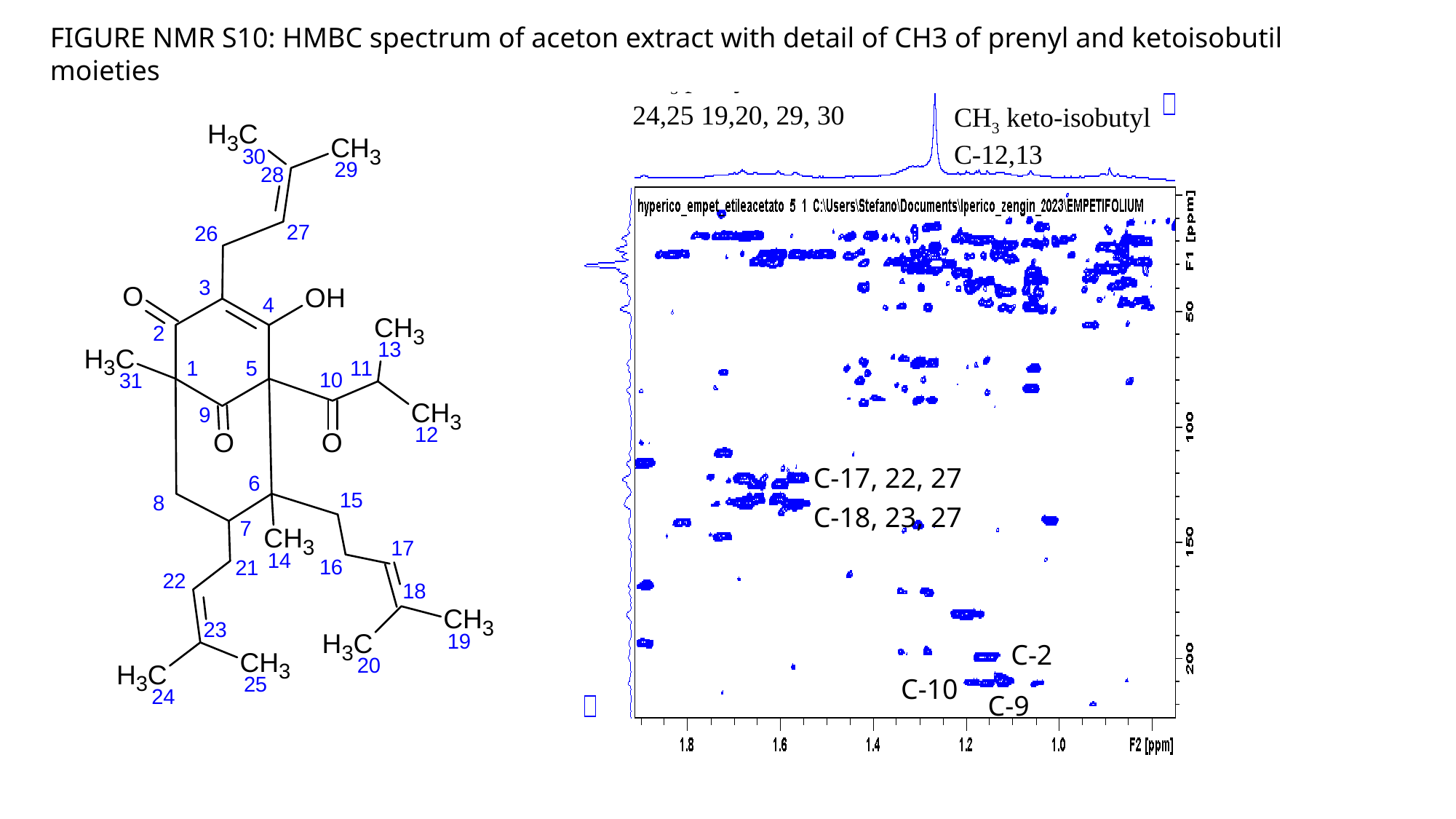

FIGURE NMR S10: HMBC spectrum of aceton extract with detail of CH3 of prenyl and ketoisobutil moieties
CH3 prenyl moieties 24,25 19,20, 29, 30
CH3 keto-isobutyl
C-12,13
C-17, 22, 27
C-18, 23, 27
C-2
C-10
C-9

## Slide 14
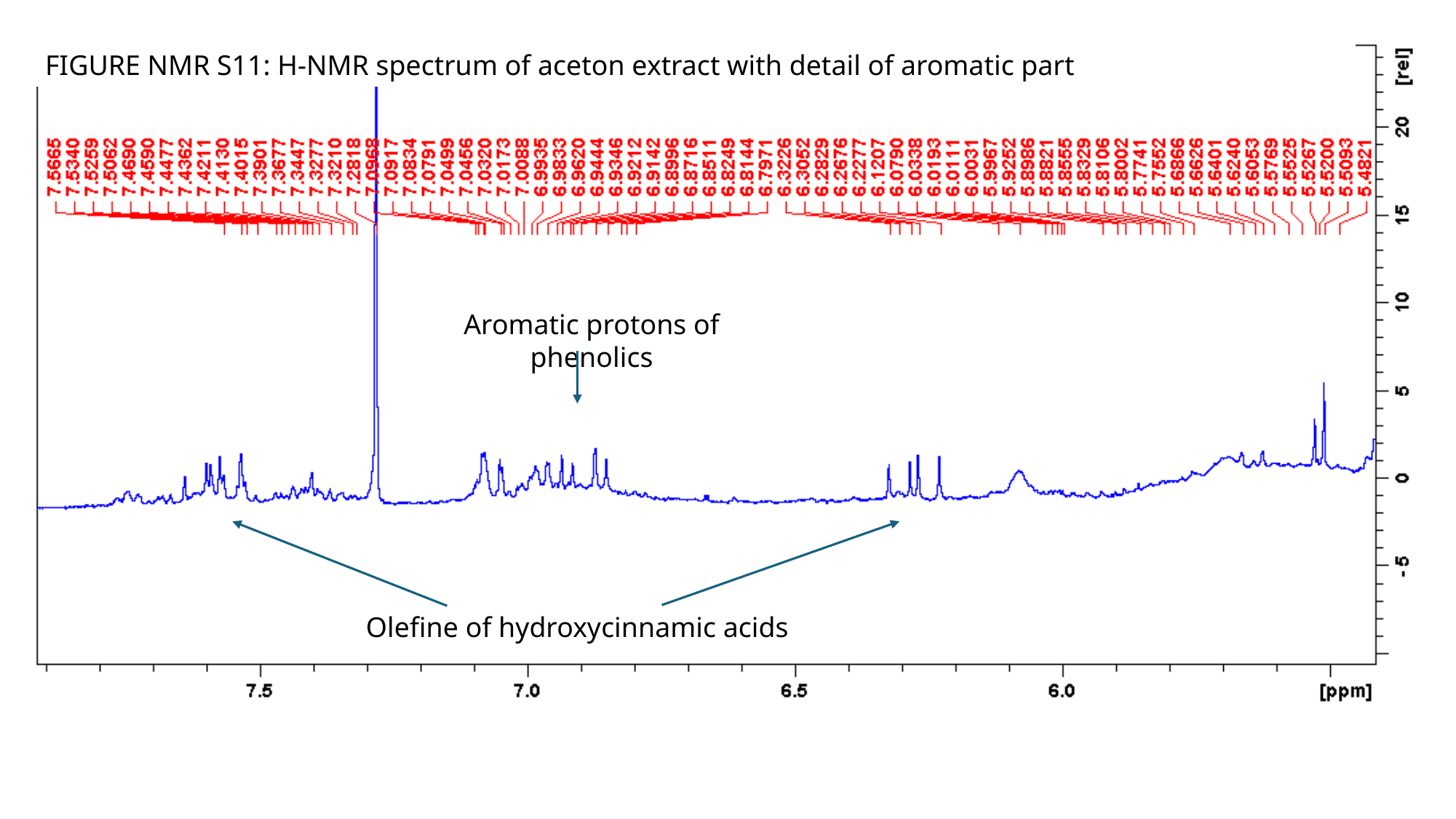

FIGURE NMR S11: H-NMR spectrum of aceton extract with detail of aromatic part
Aromatic protons of phenolics
Olefine of hydroxycinnamic acids

## Slide 15
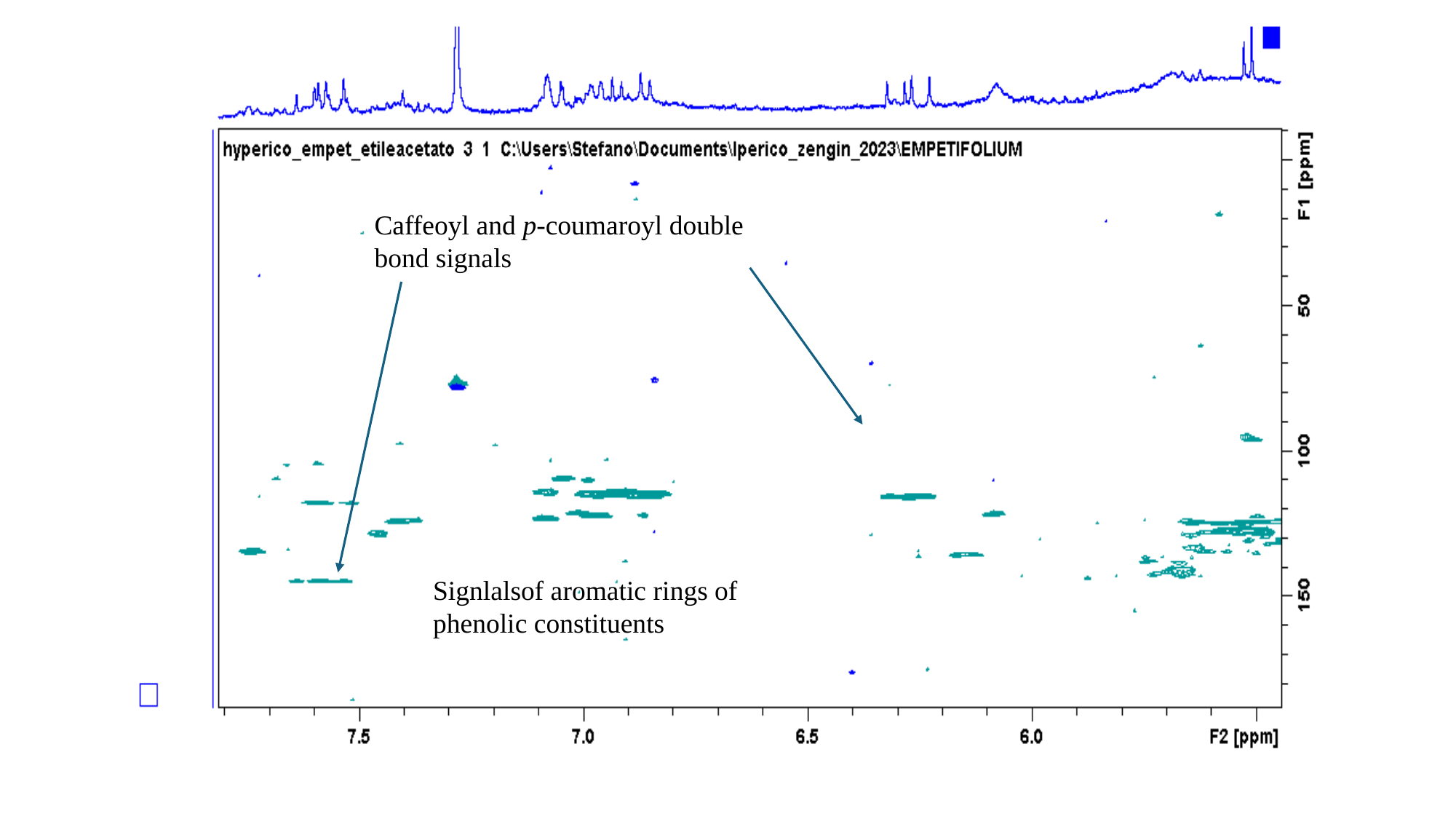

Caffeoyl and p-coumaroyl double bond signals
Signlalsof aromatic rings of phenolic constituents

## Slide 16
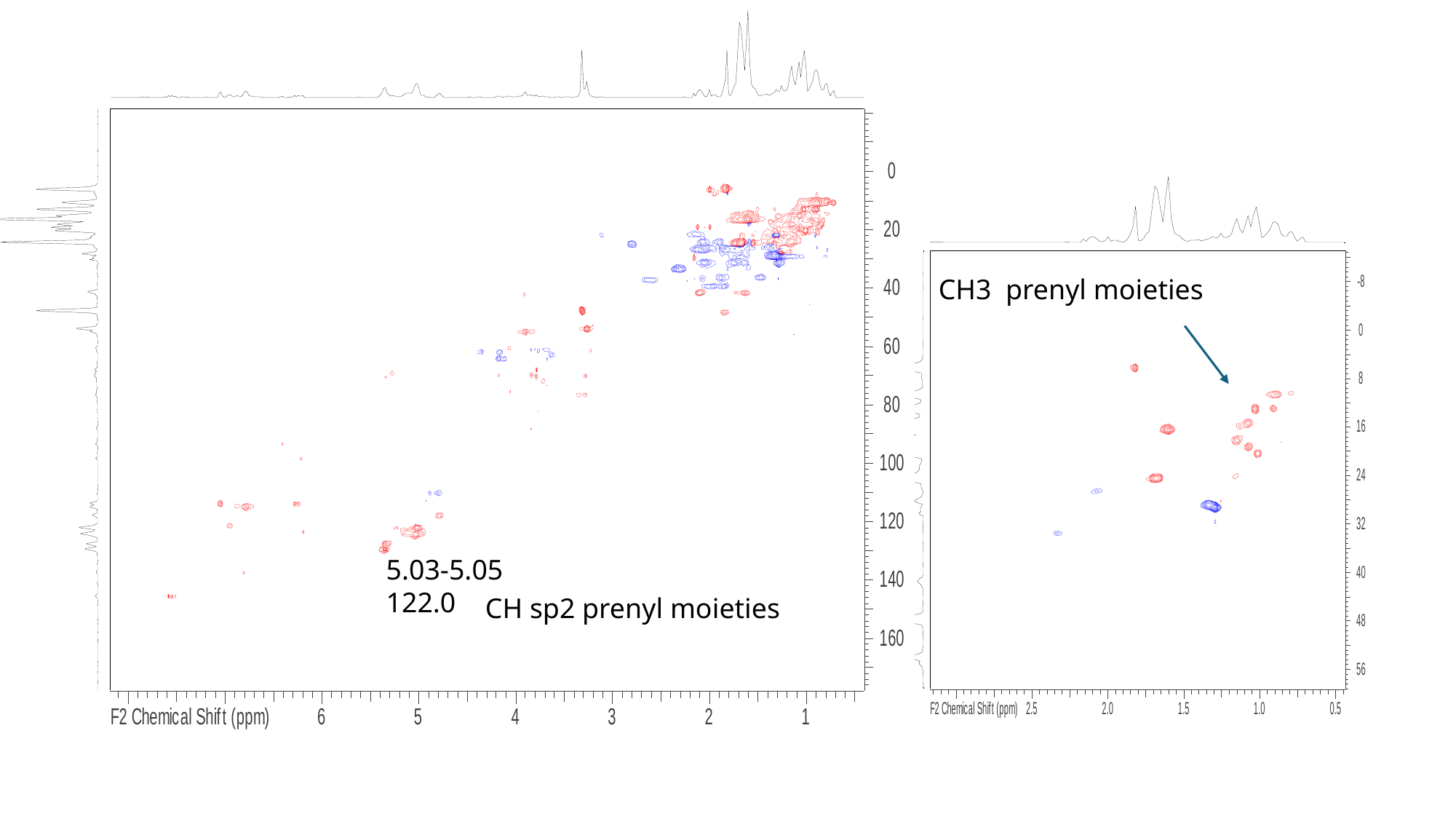

CH3 prenyl moieties
5.03-5.05
122.0
CH sp2 prenyl moieties

## Slide 17
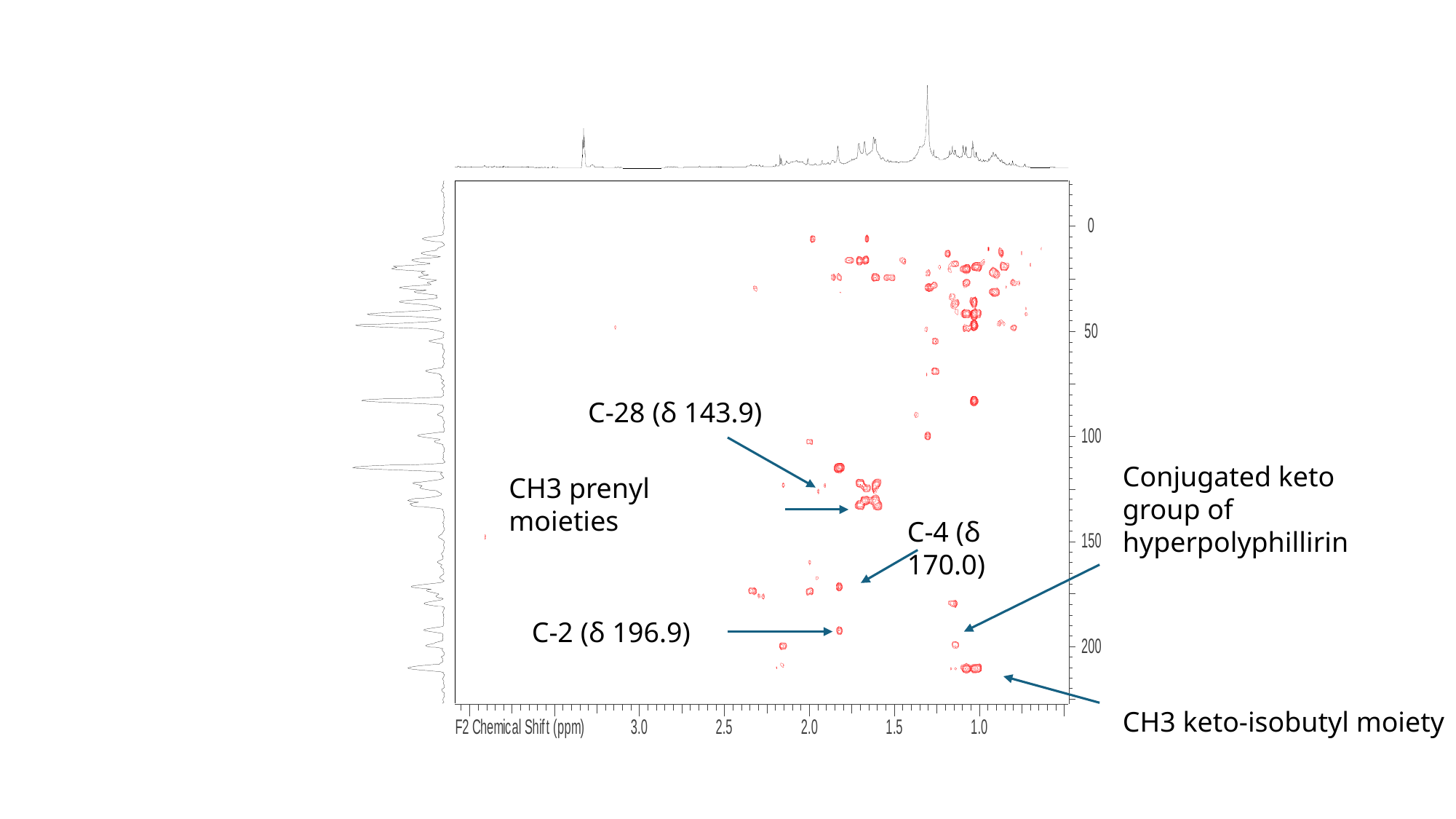

Conjugated keto group of hyperpolyphillirin
CH3 prenyl moieties
CH3 keto-isobutyl moiety
C-28 (δ 143.9)
C-4 (δ 170.0)
C-2 (δ 196.9)

## Slide 18
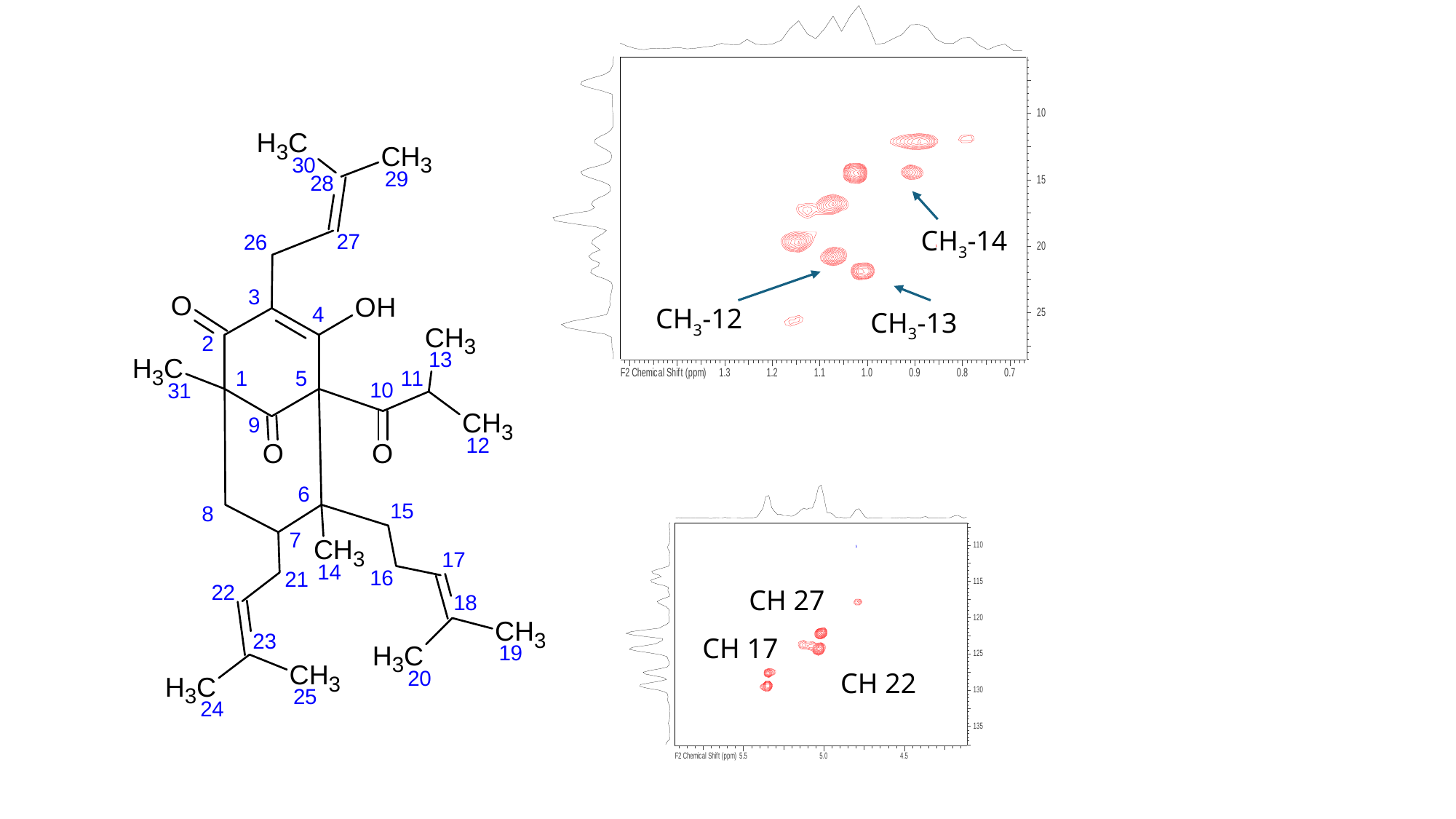

CH3-14
CH3-12
CH3-13
CH 27
CH 17
CH 22
